# Prebiotics, probiotics, and synbiotics therapy in critically ill patients: a systematic review and network meta-analysis

**DOI:** 10.1016/j.aicoj.2026.100130

**Published:** 2026-08-04

**Authors:** Junji Hatakeyama, Ryo Yamamoto, Minoru Yoshida, Kohei Yamada, Kazushige Inoue, Takayuki Irahara, Satomi Ichimaru, Nobuto Nakanishi, Naoki Higashibeppu, Kensuke Nakamura, Joji Kotani

**Affiliations:** a Advanced Critical Care and Emergency Center, Yokohama City University Medical Center, 4-57 Urafune-cho, Minami-ku, Yokohama, 232-0024, Japan; b Department of Emergency and Critical Care Medicine, Keio University School of Medicine, 35 Shinanomachi, Shinjuku, Tokyo, 160-8582, Japan; c Department of Clinical Data Management and Research, National Hospital Organization Headquarters, 2-5-21, Higashigaoka, Meguro-ku, Tokyo 152-8621, Japan; d Department of Traumatology and Critical Care Medicine, National Defense Medical College Hospital, 3-2 Namiki, Tokorozawa, Saitama, 359-0042, Japan; e Department of Critical Care Medicine and Trauma, National Hospital Organization Disaster Medical Center, 3256 Midori-cho, Tachikawa-shi, Tokyo 190-0014, Japan; f Department of Emergency and Critical Care Medicine, Aichi Medical University, 1-1, Yazakokarimata, Nagakute, Aichi 480-1195, Japan; g Food and Nutrition Service, Fujita Health University Hospital, 1-98 Dengakugakubo, Kutsukake-cho, Toyoake-shi, Aichi, Japan; h Division of Disaster and Emergency Medicine, Department of Surgery Related, Kobe University Graduate School of Medicine, 7-5-2 Kusunoki, Chuo, Kobe, Hyogo, 650-0017, Japan; i Department of Surgical Management and Department of Anesthesia NHO Kyoto Medical Center, 1-1 Mukaihata-cho, Fukakusa, Fushimi-ku, Kyoto 612-8555, Japan

**Keywords:** Prebiotics, Probiotics, Synbiotics, Gastrointestinal symptoms, Network meta-analysis

## Abstract

**Background:**

Critically ill patients frequently develop intestinal dysbiosis, leading to gastrointestinal (GI) symptoms and infectious complications. No network meta-analysis (NMA) has compared the relative effectiveness of prebiotics, probiotics, and synbiotics for reducing the risk of GI symptoms in critically ill patients.

**Objective:**

This study aimed to evaluate their comparative effects in preventing GI symptoms and improving clinical outcomes.

**Methods:**

MEDLINE, CENTRAL, and Web of Science were searched through June 2025. Eligible randomized controlled trials enrolled critically ill adults and compared prebiotics, probiotics, or synbiotics with controls (placebo or usual care). The primary outcome was GI symptom incidence. Secondary outcomes included mortality, intensive care unit (ICU) length of stay, mechanical ventilation duration, infectious complications, and adverse events. A frequentist random-effects NMA was conducted. Treatment rankings were exploratory, and certainty of evidence was not formally assessed.

**Results:**

Thirty-three randomized controlled trials involving 5,073 patients were included in the analysis of GI symptoms. Compared with control, synbiotics significantly reduced the risk of GI symptoms (risk ratio [RR] 0.52; 95% confidence interval [CI]: 0.35 to 0.77), as did prebiotics (RR 0.66; 95% CI: 0.49 to 0.87). Probiotics produced a similar directional effect, but the difference was not statistically significant (RR 0.81; 95% CI: 0.60 to 1.10). No significant differences were found between the active interventions. Subgroup analyses suggested differences according to the ICU population. Among medical intensive care patients, prebiotics were associated with a lower risk of GI symptoms than control, whereas probiotics and synbiotics were associated with a lower risk among surgical intensive care patients.

**Conclusions:**

Synbiotics and prebiotics were associated with a significantly lower risk of gastrointestinal symptoms in critically ill patients, whereas probiotics showed a similar but statistically nonsignificant effect. No active intervention was significantly superior to another. The findings suggest that the relative benefits of these interventions may differ between medical and surgical intensive care populations. Nevertheless, the results should be interpreted cautiously because certainty of evidence was not formally assessed.

PROSPERO registration number: CRD420251015729; https://www.crd.york.ac.uk/PROSPERO/view/CRD420251015729

## Introduction

Critically ill patients are particularly susceptible to intestinal dysbiosis because of antibiotic exposure, intestinal ischemia, sedation, and catecholamine use [1,2], with consequences extending beyond the gastrointestinal tract and including impaired intestinal barrier function, bacterial translocation, systemic inflammation, and increased vulnerability to nosocomial infections [3,4]. Notably, gastrointestinal dysfunction affects up to 60% of intensive care unit (ICU) patients, and approximately 50% of those receiving enteral nutrition develop symptoms of feeding intolerance, including diarrhea, vomiting, and increased gastric residual volume [5]. These symptoms frequently necessitate interruption of enteral nutrition, resulting in suboptimal nutrient delivery and potentially worsening patient outcomes [6].

Therefore, gut microbiota-modulating "biotics" have attracted considerable attention. Prebiotics are substrates selectively utilized by host microorganisms to confer health benefits [7]. Probiotics are live microorganisms that, when administered in adequate amounts, confer a health benefit to the host [8]. Moreover, synbiotics are mixtures of live microorganisms and substrates selectively utilized by host microorganisms that together confer a health benefit [9]. Collectively, when administered prophylactically, these interventions may help restore gut homeostasis, reduce the occurrence of gastrointestinal symptoms, prevent infectious complications, and potentially improve clinical outcomes in critically ill patients.

The Japanese Critical Care Nutrition Guideline 2024 (JCCNG 2024) strongly recommends prebiotics (GRADE 1B) and synbiotics (GRADE 1C), whereas probiotics are conditionally recommended (GRADE 2C) [10]. Although no difference in in-hospital mortality was observed, the guidelines reported differences in adverse events, including diarrhea. Previous network meta-analyses have assessed the effects of biotic interventions on ventilator-associated pneumonia (VAP) and nosocomial infections in critically ill patients. Nonetheless, none have systematically compared the efficacy of prebiotics, probiotics, and synbiotics regarding gastrointestinal symptoms [11,12]. Furthermore, gastrointestinal symptoms are clinically important outcomes because they directly reflect gut function, serve as the most proximal indicator of microbiome modulation, and substantially influence nutritional adequacy and patient comfort in the ICU [13,14].

Although several studies have evaluated prebiotics, probiotics, and synbiotics simultaneously [11,12], no studies have assessed gastrointestinal symptoms as an outcome in critically ill patients. As determining which biotic intervention is most effective in reducing the incidence of gastrointestinal symptoms in critically ill patients is clinically important, this study aimed to systematically identify randomized controlled trials evaluating biotics in critically ill patients and to compare the relative efficacy of each biotic group in preventing gastrointestinal symptoms using a network meta-analysis.

## Methods

The systematic review was registered in PROSPERO (CRD420251015729) and was reported in accordance with the Preferred Reporting Items for Systematic Reviews and Meta-Analyses guidelines for network meta-analyses [15].

## Search strategy

MEDLINE, the Cochrane Central Register of Controlled Trials (CENTRAL), and Web of Science were searched on June 2, 2025. No restrictions were applied to the publication date, and only studies published in English were considered eligible. Unpublished studies and conference abstracts were excluded. The detailed search strategy is displayed in eTable S1.

## Study selection and inclusion criteria

Randomized controlled trials (RCTs) enrolling critically ill adult patients (≥18 years) admitted to the ICU for any medical, surgical, or trauma-related condition were included. Eligible interventions comprised prebiotics, probiotics, or synbiotics, which were compared with the control. Control conditions were defined as placebo or usual care; enteral formulas containing dietary fiber as part of usual care were also considered controls.

## Outcomes

The primary outcome was the incidence of new-onset gastrointestinal symptoms, including diarrhea, vomiting, increased gastric residual volume, and constipation, over the study period. As these symptoms reflect distinct pathophysiological processes, diarrhea and increased gastric residual volume (GRV) or feeding intolerance were also analyzed separately. Other secondary outcomes were mortality, ICU length of stay, mechanical ventilation duration, infectious complications, and adverse events.

## Data extraction and risk of bias assessment

Three reviewers (K.I., T.I., and S.I.) independently screened titles and abstracts in the first round, followed by full-text review in the second round. Reference management and screening were performed using EndNote, version 21 (Clarivate, Philadelphia, PA, USA). Extracted data included study characteristics, patient demographics, ICU setting, details of the biotic intervention (type, dose, and duration), and outcome data for all predefined endpoints. When relevant information was unclear or unavailable in the published report, the registered study protocol was consulted. Discrepancies among reviewers were resolved through discussion and, when necessary, adjudicated by a fourth reviewer (J.H.). Risk of bias in individual studies was independently assessed using the Cochrane Risk of Bias 2 (RoB 2) tool [16], and disagreements were resolved by consensus.

## Statistical analysis

Binary outcomes were summarized as risk ratios (RRs) with 95% confidence intervals (CIs), whereas continuous outcomes were summarized as mean differences (MDs) with 95% CIs. For continuous outcomes, the mean, standard deviation (SD), and number of participants analyzed per group were extracted. When medians with ranges or interquartile ranges were reported, these summary statistics were converted to means and SDs using validated methods to enable inclusion in the meta-analyses [17,18].

A frequentist network meta-analysis was conducted using a random-effects model as the primary analytical approach. Placebo and usual care were combined into a single control node. Global inconsistency was evaluated using the design-by-treatment interaction model, whereas local inconsistency was assessed using the node-splitting approach. The probability of each intervention ranking as the most effective for each outcome was estimated, and treatment rankings were summarized using the surface under the cumulative ranking curve (SUCRA). Conventional pairwise meta-analyses were also performed for all available direct comparisons to summarize direct evidence and support the assessment of consistency between direct and indirect estimates. For outcomes with a sufficient number of studies, comparison-adjusted funnel plots were constructed to explore potential small-study effects. Funnel plot asymmetry was additionally evaluated using Egger’s regression test applied to the comparison-adjusted effect sizes. All analyses were performed using Stata/SE (version 17.0; StataCorp, College Station, TX, USA) with the network meta-analysis command suite [19]. Statistical significance was set at p < 0.05.

## Subgroup and sensitivity analyses

Prespecified subgroup analyses were undertaken to explore potential sources of clinical heterogeneity. Subgroups were defined according to patient population (medical, surgical, or mixed), intervention duration, and whether oligosaccharides were included as a component of the intervention. Intervention duration was obtained from the methods section of each study or, when not explicitly reported, from the mean or median treatment duration presented in the results. When sufficient data were available, subgroup analyses were performed within the network meta-analytic framework.

Several sensitivity analyses were conducted to assess the robustness of the primary findings. These analyses comprised the exclusion of studies judged to be at high risk of bias using the RoB 2 tool, the exclusion of studies with fewer than 50 participants to assess the potential influence of small-study effects, and restricting the analysis to trials with a limited control group, defined as placebo or usual care clearly confirmed to be fiber-free. Subsequently, sensitivity analysis results were compared with those of the primary analysis to evaluate the consistency of the findings.

## Results

**Fig. 1:**
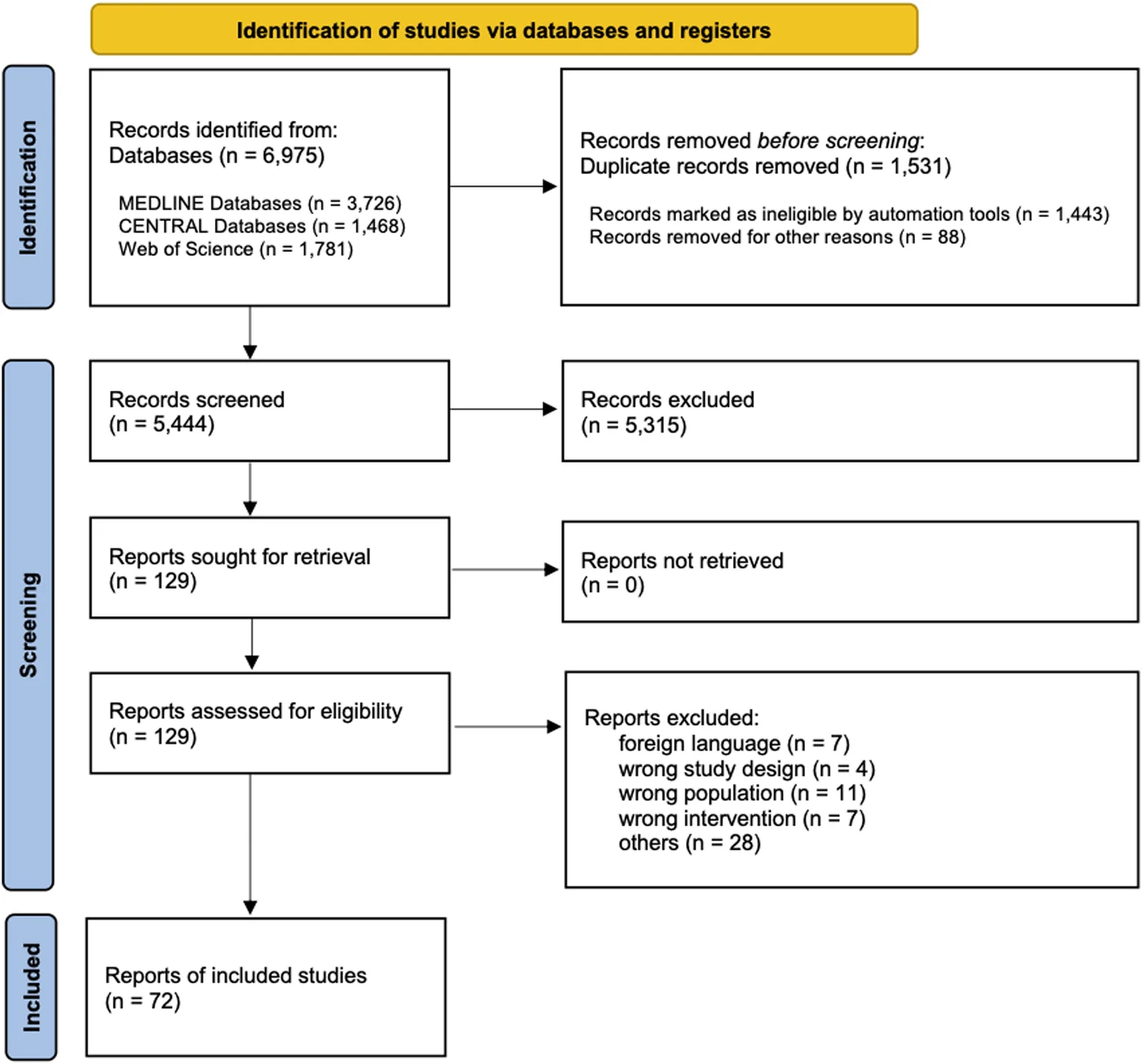
Study selection.

The database search identified 6,975 records across three databases: MEDLINE (n = 3,726), CENTRAL (n = 1,468), and Web of Science (n = 1,781). After 1,531 duplicates were removed, 5,444 records underwent title and abstract screening. Ultimately, 72 studies were included in this systematic review and network meta-analysis, with the study selection process displayed in Fig. 1. Overall, 8,728 patients were enrolled across the 72 included studies. Probiotics, prebiotics, and synbiotics were evaluated in 32, 20, and 20 studies, respectively. Placebo and usual care were used as comparators in 42 and 24 studies, respectively. The characteristics of the included studies are summarized in Table 1. Risk of bias assessments for the 33 studies contributing to the network meta-analysis of gastrointestinal symptoms are presented in eFig. S1.

**Table 1:** Characteristics of studies included in the network meta-analysis.

Study (year)	Country	ICU population	Setting	Sample size (n)	Intervention	Control	Duration of intervention	Oligosaccharides included	Risk of bias for GI symptoms
Mahmoodpoor 2023	Iran	Surgical	Single-center	40	Probiotics	Placebo	7–13 days	No	Low
Johnstone 2021	Mainly Canada	Mixed	Multicenter	2650	Probiotics	Placebo	7–13 days	No	Low
Anandaraj 2019	India	Medical	Single-center	146	Probiotics	Placebo	< 7 days	No	Not assessed
Wongseree 2023	Thailand	Medical	Single-center	24	Synbiotics	Usual care	7–13 days	Yes	Some concerns
Cook 2016	Canada and USA	Mixed	Multicenter	150	Probiotics	Placebo	≥ 14 days	No	Not assessed
Habib 2020	Egypt	Trauma	Single-center	65	Probiotics	Placebo	≥ 14 days	No	Not assessed
Prasoon 2022	India	Mixed	Single-center	120	Probiotics	Usual care	Not reported	No	High
Aghababaee 2024	Iran	Medical	Single-center	70	Synbiotics	Placebo	7–13 days	Yes	Not assessed
Nazari 2020	Iran	Trauma	Multicenter	150	Probiotics	Placebo	Not reported	No	Not assessed
Ataollahi 2025	Iran	Mixed	Single-center	40	Probiotics	Placebo	≥ 14 days	No	Not assessed
Tan 2011	China	Trauma	Single-center	52	Probiotics	Usual care	≥ 14 days	No	Not assessed
Zeng 2016	China	Mixed	Multicenter	250	Probiotics	Usual care	7–13 days	No	Not assessed
Kooshki 2018	Iran	Mixed	Multicenter	60	Prebiotics	Usual care	Not reported	No	High
Rohith 2023	India	Medical	Single-center	76	Synbiotics	Placebo	≥ 14 days	No	Low
Morrow 2010	USA	Mixed	Multicenter	138	Probiotics	Prebiotics	Not reported	No	Low
Chen 2021	China	Medical	Single-center	46	Prebiotics	Usual care	Not reported	No	Some concerns
Abbaszadeh 2024	Iran	Trauma	Multicenter	46	Probiotics	Placebo	≥ 14 days	No	Not assessed
Park 2025	USA	Medical	Single-center	90	Prebiotics	Placebo	7–13 days	No	Some concerns
Gatt 2010	UK	Surgical	Single-center	50	Synbiotics	Placebo	≥ 14 days	Yes	Some concerns
Yagmurdur 2016	Turkey	Medical	Single-center	120	Prebiotics	Usual care	< 7 days	Yes	Some concerns
Tsilika 2022	Greece	Trauma	Multicenter	112	Probiotics	Placebo	≥ 14 days	No	Low
Lu 2024	China	Mixed	Single-center	24	Probiotics	Usual care	Not reported	No	High
McNaught 2005	UK	Mixed	Single-center	103	Probiotics	Usual care	7–13 days	No	Not assessed
Spindler-Vesel 2007	Slovenia	Trauma	Single-center	132	Prebiotics	Synbiotics	≥ 14 days	No	Some concerns
Tzikos 2022	Greece	Trauma	Multicenter	112	Probiotics	Placebo	≥ 14 days	No	Not assessed
Dehghani 2023	Iran	Surgical	Single-center	105	Synbiotics	Usual care	7–13 days	Yes	Some concerns
Xi 2017	China	Mixed	Single-center	166	Prebiotics	Usual care	< 7 days	No	Some concerns
Barraud 2010	France	Medical	Single-center	167	Probiotics	Placebo	≥ 14 days	No	Low
Abbasi 2023	Iran	Trauma	Multicenter	80	Synbiotics	Placebo	7–13 days	Yes	Not assessed
Seifi 2022a	Iran	Medical	Single-center	42	Synbiotics	Placebo	7–13 days	Yes	Not assessed
Seifi 2022b	Iran	Medical	Single-center	38	Synbiotics	Placebo	7–13 days	Yes	Not assessed
Wang 2021	China	Medical	Single-center	61	Probiotics	Placebo	Not reported	No	Some concerns
Seifi 2022	Iran	Medical	Single-center	38	Synbiotics	Placebo	7–13 days	Yes	Some concerns
Malik 2016	Malaysia	Mixed	Single-center	60	Probiotics	Placebo	7–13 days	No	Not assessed
Litton 2021	Australia	Mixed	Multicenter	221	Probiotics	Placebo	≥ 14 days	No	Not assessed
Giamarellos-Bourboulis 2009	Greece	Surgical	Multicenter	72	Synbiotics	Placebo	≥ 14 days	No	Not assessed
Alberda 2007	Canada	Mixed	Single-center	28	Prebiotics	Synbiotics	7–13 days	Yes	Not assessed
Majid 2014	UK	Mixed	Multicenter	47	Prebiotics	Placebo	7–13 days	Yes	High
Ferrie 2011	Australia	Mixed	Single-center	36	Synbiotics	Prebiotics	7–13 days	No	Some concerns
Shimizu 2018	Japan	Mixed	Multicenter	77	Synbiotics	Usual care	≥ 14 days	Yes	Some concerns
Mahmoodpoor 2019	Iran	Surgical	Multicenter	120	Probiotics	Placebo	≥ 14 days	No	Some concerns
van der Spoel 2007	Netherlands	Mixed	Multicenter	308	Prebiotics	Placebo	< 7 days	No	Low
Jain 2004	UK	Surgical	Single-center	90	Synbiotics	Placebo	7–13 days	Yes	Not assessed
Chittawatanarat 2010	Thailand	Surgical	Single-center	34	Prebiotics	Usual care	7–13 days	Yes	Some concerns
Rushdi 2004	Egypt	Mixed	Single-center	30	Prebiotics	Usual care	< 7 days	No	High
Tuncay 2018	Turkey	Medical	Single-center	68	Prebiotics	Usual care	≥ 14 days	Yes	High
Knight 2009	UK	Mixed	Single-center	300	Synbiotics	Placebo	≥ 14 days	No	Some concerns
Frohmader 2010	Australia	Mixed	Single-center	45	Probiotics	Placebo	7–13 days	No	Some concerns
Kotzampassi 2006	Greece	Trauma	Multicenter	65	Synbiotics	Placebo	≥ 14 days	No	Not assessed
Kwon 2015	USA	Medical	Single-center	70	Probiotics	Usual care	7–13 days	No	Not assessed
Forestier 2008	France	Mixed	Single-center	208	Probiotics	Placebo	< 7 days	No	Not assessed
Caparrós 2001	Spain	Mixed	Multicenter	220	Prebiotics	Usual care	≥ 14 days	Yes	Some concerns
Dobb 1990	Australia	Mixed	Single-center	91	Prebiotics	Usual care	≥ 14 days	No	High
Falcão de Arruda 2004	Brazil	Trauma	Single-center	20	Probiotics	Usual care	7–13 days	No	Not assessed
Koutelidakis 2010	Greece	Trauma	Multicenter	65	Synbiotics	Placebo	≥ 14 days	No	Not assessed
Schultz 2000	USA	Mixed	Single-center	44	Prebiotics	Placebo	< 7 days	No	High
Karakan 2007	Turkey	Medical	Single-center	30	Prebiotics	Usual care	7–13 days	Not reported	Not assessed
Lee 2016	South Korea	Mixed	Single-center	22	Prebiotics	Usual care	7–13 days	No	Not assessed
Spapen 2001	Belgium	Medical	Single-center	25	Prebiotics	Usual care	7–13 days	No	High
Fazilaty 2018	Iran	Trauma	Single-center	40	Prebiotics	Placebo	≥ 14 days	No	Not assessed
Bleichner 1997	France	Mixed	Multicenter	128	Probiotics	Placebo	7–13 days	No	Some concerns
Klarin 2008	Sweden	Mixed	Multicenter	44	Probiotics	Placebo	< 7 days	No	Not assessed
Klarin 2005	Sweden	Mixed	Single-center	15	Probiotics	Usual care	7–13 days	No	Not assessed
Sanaie 2014	Iran	Mixed	Single-center	40	Probiotics	Placebo	7–13 days	No	Not assessed
Naslowski 2025	Brazil	Mixed	Single-center	70	Synbiotics	Placebo	7–13 days	Yes	High
Ebrahimi-Mameghani 2013	Iran	Surgical	Single-center	40	Probiotics	Placebo	7–13 days	No	Not assessed
Kasiri 2023	Iran	Mixed	Single-center	80	Synbiotics	Placebo	≥ 14 days	Yes	Some concerns
Sanaie 2013	Iran	Mixed	Single-center	40	Probiotics	Placebo	7–13 days	No	Not assessed
Kamel 2021	Egypt	Trauma	Single-center	85	Probiotics	Usual care	< 7 days	No	Not assessed
Rayes 2002	Germany	Surgical	Single-center	95	Synbiotics	Prebiotics	7–13 days	No	Some concerns
Vahdat Shariatpanahi 2018	Iran	Mixed	Single-center	32	Prebiotics	Placebo	7–13 days	Yes	High
Mao 2022	China	Medical	Single-center	60	Synbiotics	Probiotics	7–13 days	No	High

Summary of the key characteristics of the randomized controlled trials included in the network meta-analysis, including the study setting, patient population, interventions (prebiotics, probiotics, synbiotics, usual care, or placebo), sample size, follow-up duration, and gastrointestinal outcomes assessed. Risk of bias was evaluated using the Cochrane RoB 2 tool for trials that reported the primary outcome (gastrointestinal symptoms) only; studies that did not report gastrointestinal symptoms were marked as “Not assessed”.

*GI* Gastrointestinal*, ICU* Intensive Care Unit, *UK* United Kingdom of Great Britain, Northern Ireland, *USA* United States of America.

### Primary outcome: gastrointestinal symptoms

**Fig. 2:**
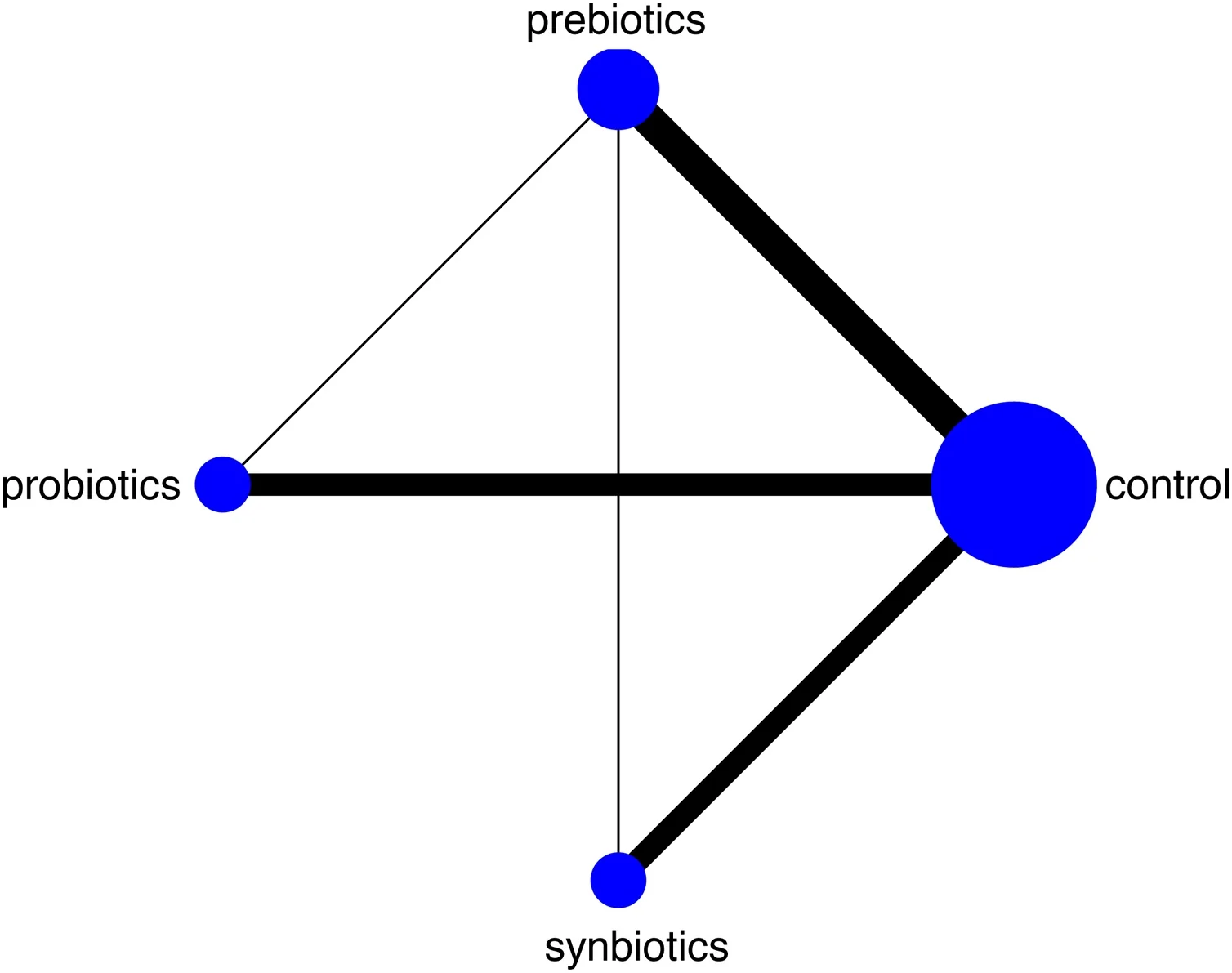
Network map for gastrointestinal symptoms. Overall, 33 studies were included in the network. Each node represents a treatment, and node size is proportional to the number of randomized patients. Line thickness is proportional to the number of studies contributing to each comparison.

**Fig. 3:**
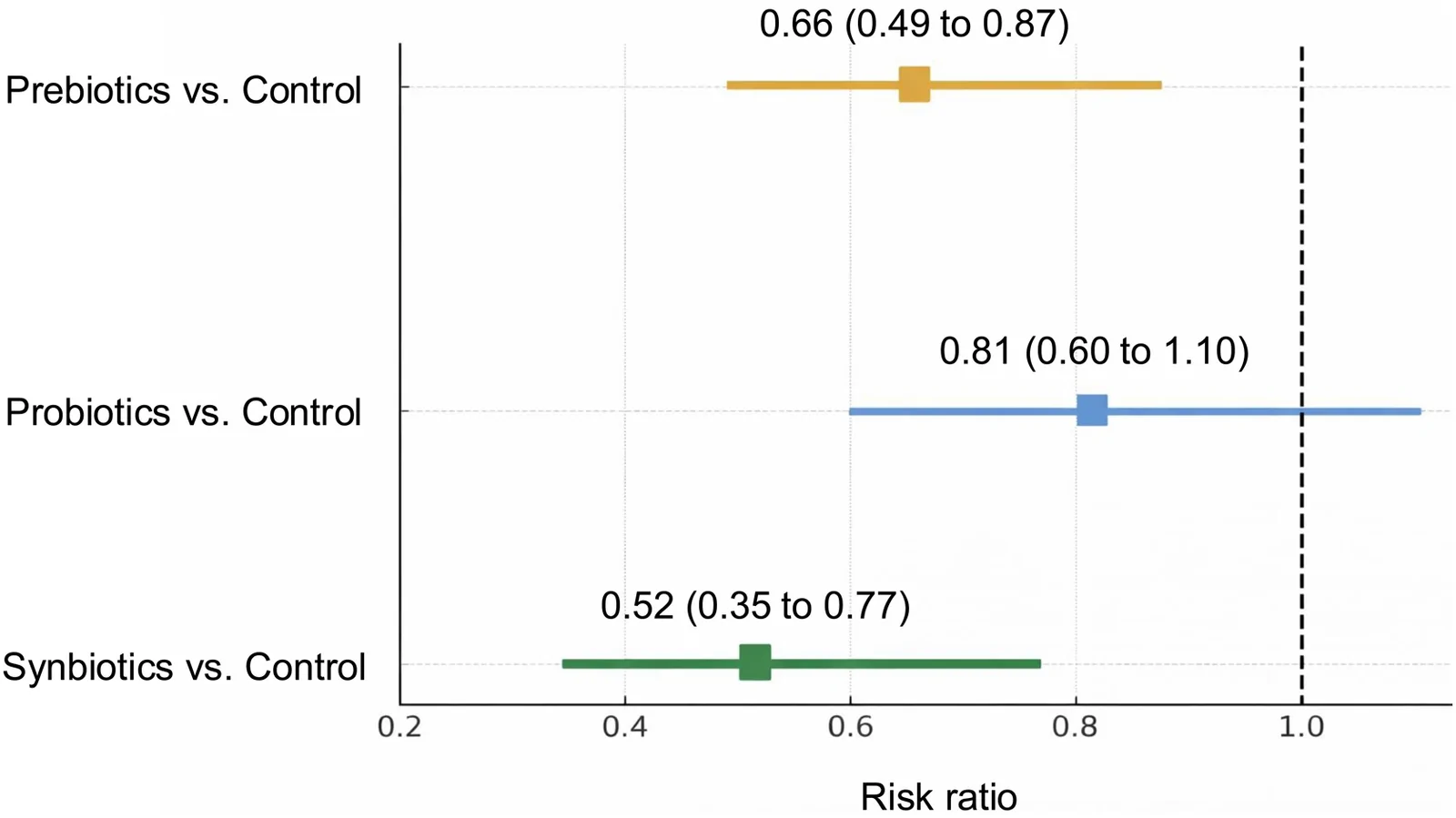
Forest plot of network meta-analysis for gastrointestinal symptoms: prebiotics, probiotics, and synbiotics vs control. Forest plot comparing prebiotics, probiotics, and synbiotics with controls for gastrointestinal symptoms. The results were expressed as risk ratios with 95% confidence intervals (CIs).

Overall, 33 studies enrolling 5,073 patients reported gastrointestinal symptoms and were included in the network meta-analysis of the primary outcome. Study-level event counts are presented in eTable S2. The network comprised four nodes representing the control, prebiotic, probiotic, and synbiotic groups, all connected within a single network map, which permitted valid indirect comparisons across treatments (Fig. 2). In the network meta-analysis, synbiotics and prebiotics significantly reduced the risk of gastrointestinal symptoms compared with control (synbiotics: risk ratio [RR] 0.52; 95% confidence interval [CI]: 0.35 to 0.77 and prebiotics: RR 0.66; 95% CI: 0.49 to 0.87), whereas probiotics showed a directionally similar but statistically nonsignificant estimate (RR 0.81; 95% CI: 0.60 to 1.10) (Fig. 3). No statistically significant differences were observed among the active interventions (Table 2). Exploratory SUCRA rankings are presented in eTable S3. Conventional pairwise meta-analyses yielded results consistent with the network estimates (eTable S4), and neither global nor local inconsistencies were detected (eTables S5 and S6). However, Egger’s regression test using comparison-adjusted effect sizes suggested potential small-study effects (p = 0.035) (eFig. S2). For diarrhea, 27 RCTs involving 4,786 patients were included. Prebiotics and synbiotics were associated with a lower risk than control (prebiotics: RR 0.65; 95% CI: 0.48 to 0.88, synbiotics: RR 0.52; 95% CI: 0.31 to 0.87), whereas probiotics were not (RR 0.84; 95% CI: 0.61 to 1.14). For increased GRV or feeding intolerance, nine RCTs involving 826 patients were included. Prebiotics and probiotics were associated with a lower risk than control (prebiotics: RR 0.61; 95% CI: 0.48 to 0.79, probiotics: RR 0.51; 95% CI: 0.31 to 0.84), whereas synbiotics were not (RR 0.84; 95% CI: 0.51 to 1.40). No statistically significant differences were observed among the active interventions in either symptom-specific analysis.

**Table 2:** League table of network meta-analysis for gastrointestinal symptoms.

	control	prebiotics	probiotics	synbiotics
control	–	0.66 (0.49, 0.87)	0.81 (0.60, 1.10)	0.52 (0.35, 0.77)
prebiotics	1.52 (1.15, 2.03)	–	1.24 (0.89, 1.74)	0.79 (0.50, 1.24)
probiotics	1.23 (0.91, 1.66)	0.81 (0.57, 1.13)	–	0.63 (0.39, 1.02)
synbiotics	1.94 (1.31, 2.88)	1.27 (0.81, 2.00)	1.58 (0.98, 2.56)	–

League table summarizing the relative effects of prebiotics, probiotics, and synbiotics compared with the control on gastrointestinal symptoms, as estimated using a random-effects network meta-analysis. Relative effects are presented as risk ratios (RRs) with 95% confidence intervals. Each cell represents the comparison between the treatment listed in the row and that listed in the column. RRs <1 indicate a lower risk of gastrointestinal symptoms with the column treatment compared with the row treatment.

### Secondary outcomes

The numbers of RCTs and patients contributing to each secondary outcome were as follows: mortality, 43 RCTs (6,064 patients); ICU mortality, 20 RCTs (4,698 patients); ICU length of stay, 37 RCTs (6,109 patients); duration of mechanical ventilation, 21 RCTs (4,668 patients); infectious complications, 45 RCTs (6,624 patients); VAP, 22 RCTs (5,032 patients); and adverse events, 15 RCTs (3,876 patients). The network map for each outcome is shown in eFig. S3. Conversion from medians to means was required for 4 of the 37 studies reporting ICU length of stay and 1 of the 21 studies reporting duration of mechanical ventilation. The same validated conversion methods were applied consistently to all studies requiring conversion.

### Mortality

In the network meta-analysis, none of the interventions demonstrated a statistically significant reduction in mortality compared with the control: prebiotics (RR 0.79; 95% CI: 0.62 to 1.02), probiotics (RR 0.94; 95% CI: 0.85 to 1.04), and synbiotics (RR 0.83; 95% CI: 0.66 to 1.05) (eFigure S4). No significant global or local inconsistencies were detected, and the comparison-adjusted funnel plot showed no marked asymmetry (eTables S5 and S6; eFigure S5). Regarding ICU mortality, the trend was similar to that of mortality.

### Length of ICU stay

Prebiotics (MD -1.70 days; 95% CI: −3.27 to −0.14) and probiotics (MD −1.52 days; 95% CI: −2.75 to −0.29) significantly reduced ICU length of stay compared with control, whereas synbiotics did not (MD −1.48 days; 95% CI: −3.11 to 0.16) (eFigure S4). No global or local inconsistency was detected (eTables S5 and S6).

### Mechanical ventilation duration

Synbiotics significantly shortened mechanical ventilation duration relative to control (MD, -2.73 days; 95% CI: -5.43 to -0.03), whereas prebiotics (MD, -2.18 days; 95% CI: -5.39 to 1.02) and probiotics (MD, -1.03 days; 95% CI: -2.91 to 0.86) did not reach statistical significance (eFigure S4). No global or local inconsistency was detected, and the comparison-adjusted funnel plot demonstrated no marked asymmetry (eTables S5 and S6; eFigure S5).

### Infectious complications

Significant global inconsistency was detected in the network meta-analysis using the design-by-treatment interaction model (p = 0.02). Local inconsistency was also identified in the node-splitting analyses for the comparisons of prebiotics versus control and synbiotics versus control. In the direct pairwise meta-analyses, prebiotics, probiotics, and synbiotics were each associated with a lower risk of infectious complications than control (prebiotics: RR 0.62; 95% CI: 0.42 to 0.91, probiotics: RR 0.64; 95% CI: 0.51 to 0.79, synbiotics: RR 0.65; 95% CI: 0.48 to 0.87, respectively).

For VAP, probiotics (RR 0.64; 95% CI: 0.47 to 0.85) and synbiotics (RR 0.59; 95% CI: 0.37 to 0.94) were also associated with a lower risk than control; conversely, prebiotics were not (RR 0.85; 95% CI: 0.45 to 1.60) (eFigure S4). No significant differences were observed among the active interventions (eTable S7). No evidence of global or local inconsistency was detected, and the comparison-adjusted funnel plot showed no marked asymmetry (eTables S5 and S6; eFigure S5).

### Adverse events

No significant differences in adverse events were observed between the intervention and control groups, although the estimates were imprecise (eFigure S4). Detailed results for all secondary outcomes are provided in the Supplementary Material.

### Subgroup and sensitivity analyses

In the prespecified subgroup and sensitivity analyses conducted within the random-effects network meta-analytic framework, treatment effects and corresponding SUCRA rankings varied across clinical contexts. Among surgical ICU patients, probiotics and synbiotics were preferred over controls for reducing gastrointestinal symptoms, whereas prebiotics were not. Conversely, among medical ICU patients, prebiotics were favored over control; additionally, probiotics and synbiotics showed no clear benefit. Regarding intervention duration, the evidence was largely concentrated in the 7–13-day treatment range; within this subgroup, synbiotics remained beneficial compared with control, whereas prebiotics and probiotics were not. In trials incorporating oligosaccharide supplementation, both prebiotics and synbiotics were favored over control, with no clear distinction between them. In the sensitivity analysis excluding studies judged to be at high risk of bias, synbiotics were associated with a lower risk of gastrointestinal symptoms than control, whereas prebiotics were not. However, significant global inconsistency was detected in this restricted network (p = 0.04), with evidence of local inconsistency for the comparisons of prebiotics versus control and probiotics versus control (eTables S5 and S6). Another sensitivity analysis excluding studies with fewer than 50 participants included 21 RCTs involving 4,678 participants. No evidence of funnel plot asymmetry was detected (Egger’s test, p = 0.19; eFigure S8). In this analysis, synbiotics were associated with a significantly lower risk of gastrointestinal symptoms than control (RR 0.53; 95% CI, 0.36 to 0.76), whereas prebiotics and probiotics were not (eFigure S7). In contrast to the primary analysis, statistically significant differences were observed between synbiotics and the other active interventions. Exploratory treatment rankings placed synbiotics highest, with a SUCRA value of 1.00, followed by prebiotics (0.50), probiotics (0.30), and control (0.10) (eTable S3). In the sensitivity analysis restricted to trials with a limited control group (28 RCTs, 4,723 patients), the findings were similar to those of the primary analysis. Detailed results of subgroup and sensitivity analyses are provided in the Supplementary Material (eFigures S6–S8 and eTable S7).

## Discussion

In this systematic review and network meta-analysis comparing the effects of prebiotics, probiotics, and synbiotics on gastrointestinal symptoms and clinical outcomes in critically ill patients, synbiotics and prebiotics were associated with a lower risk of gastrointestinal symptoms than control, whereas probiotics showed a directionally similar but statistically nonsignificant effect. No statistically significant differences were observed among the active interventions. On note, analyses on individual GI symptom suggested different effects between types of biotics, while these results should not be interpreted as demonstrating comparative superiority among them.

Although no significant differences were observed among the active interventions, the association between synbiotic use and a lower risk of gastrointestinal symptoms compared with control is biologically plausible. Synbiotics combine live microorganisms with substrates that promote microbial growth and metabolic activity [[7], [8], [9]], potentially helping to counteract dysbiosis and the depletion of short-chain fatty acids in critically ill patients [20,21]. Supporting this biological rationale, an RCT in patients with sepsis reported increased fecal counts of Bifidobacterium and Lactobacillus and higher short-chain fatty acid concentrations following synbiotic administration [22].

Differential effects of biotic interventions on clinical outcomes were observed. Although synbiotics and prebiotics were associated with a lower risk of gastrointestinal symptoms than control, probiotics and synbiotics were associated with a lower risk of VAP. Notably, this discrepancy may reflect distinct mechanisms of action. Specifically, prebiotics primarily modulate gut microbiome composition and SCFA production, which may more directly influence gastrointestinal function by enhancing intestinal barrier integrity and motility [23,24]. Conversely, the associations of probiotics and synbiotics with a lower VAP risk may be partly explained by the gut–lung axis. Through this pathway, critical illness–related dysbiosis and disruption of the intestinal barrier may promote bacterial translocation, systemic inflammation, and impaired pulmonary host defense [[25], [26], [27]]. A randomized controlled trial in patients with sepsis demonstrated that synbiotic administration maintained beneficial gut flora and significantly increased SCFA levels, along with reduced VAP incidence [22]. This finding is also consistent with prior pairwise meta-analytic evidence showing that synbiotics, but not probiotics alone, were associated with a significant reduction in VAP incidence [28].

Further, subgroup analyses indicated heterogeneous treatment effects across patient populations and intervention characteristics. Probiotics and synbiotics were associated with a lower incidence of gastrointestinal symptoms in surgical ICU patients, whereas significant benefits in medical ICU patients were observed only with prebiotics. Importantly, these differences may reflect variation in baseline dysbiosis or underlying pathophysiology, although the optimal biotic approach for each population remains uncertain and requires further investigation [4,29,30]. Most included trials assessed 7–13 days of treatment, during which synbiotics remained beneficial. Nonetheless, the optimal biotic therapy duration warrants additional study. In trials incorporating oligosaccharide supplementation, both prebiotics and synbiotics significantly reduced the incidence of gastrointestinal symptoms, suggesting that specific prebiotic components may be key determinants of efficacy.

Data on gastrointestinal symptoms suitable for the primary NMA were available in only 33 of the 72 included RCTs, likely reflecting the historical focus of ICU biotics trials and previous meta-analyses on infectious outcomes, particularly VAP, and the lack of standardized gastrointestinal outcome definitions [31,32]. Compared with the previous Bayesian NMA, the present study used a frequentist framework, compared individual biotic classes with placebo or usual care rather than broader nutritional strategies, focused on gastrointestinal symptoms rather than VAP prevention, and included 72 rather than 31 RCTs [12]. The two studies therefore address distinct clinical questions through different network structures, with the present work offering a larger evidence base focused on gastrointestinal function in critically ill patients. Strain- and fiber-specific effects could not be assessed because of heterogeneity in the formulations used across trials. Given that probiotic effects are strain-specific and that prebiotic fibers may differ in their effects on the gut microbiota and SCFA production, pooling diverse formulations may obscure clinically relevant effects [33]. The lower risk observed with oligosaccharide-containing regimens further suggests that intervention composition may influence efficacy, supporting future trials of well-characterized biotic formulations using standardized gastrointestinal outcomes.

This study has some limitations. Definitions and assessment methods for gastrointestinal symptoms, probiotic formulations, and prebiotic fiber types varied across studies, contributing to clinical heterogeneity and limiting recommendations regarding optimal regimens [34,35]. Direct comparisons between active interventions were sparse, and most estimates relied on indirect evidence through the control node [36]. Several studies had some concerns or a high risk of bias, and sensitivity analyses excluding high-risk studies did not fully support the primary findings, particularly for prebiotics. Significant inconsistency was also detected for infectious complications. In addition, the search excluded several databases, trial registries, and non-English full-text articles, potentially introducing publication and language bias. Estimates for ICU length of stay and mechanical ventilation duration should be interpreted cautiously because these outcomes are typically right-skewed, despite the use of validated methods to convert medians to means. Finally, certainty of evidence was not formally assessed using the GRADE framework. Given the risk of bias, small-study effects, sparse direct evidence, clinical heterogeneity, and absence of significant differences between active interventions, the findings and treatment rankings should be interpreted cautiously and should not independently inform clinical guidelines.

## Conclusion

Synbiotics and prebiotics were associated with a lower risk of gastrointestinal symptoms than control, whereas probiotics showed a directionally similar but statistically nonsignificant effect, with no significant differences among active interventions. Subgroup analyses suggested that treatment effects may vary by ICU population, but these findings should be interpreted cautiously because certainty of evidence was not formally assessed and no hierarchy of superiority among biotic classes was established.

## Authors’ contributions

J.H., M.Y., and R.Y. designed research; K.I., T.I., S.I., and J.H. conducted research; J.H. and M.Y. analyzed data and performed statistical analysis; R.Y. and K.Y. provided analytical guidance; J.H. and R.Y. wrote the paper, with input from M.Y., K.Y., N.N., N.H., K.N., and J.K.; J.H. and R.Y. had primary responsibility for final content. All authors read and approved the final manuscript.

## Consent for publication

Not applicable.

## Ethics approval and consent to participate

Not applicable.

## Declaration of Generative AI and AI-assisted technologies in the writing process

During the preparation of this work, Claude (Anthropic) was used to improve the English language and readability. The tool was not used for data analysis, interpretation of the results, or any other aspect of the research process. After use of the tool, the content was reviewed and edited as needed, and the authors take full responsibility for the content of the manuscript.

## Funding

This study did not receive any specific grants from funding agencies in the public, commercial, or non-profit sectors.

## Availability of data and material

Data described in the manuscript, codebook, and analytic code will be made available upon request to the corresponding author.

## Data availability

The data that has been used is confidential.

Data will be made available on request.

## Declaration of competing interest

The authors declare that they have no competing interests.
