# Supplementary material for: Prebiotics, probiotics, and synbiotics therapy in critically ill patients: a systematic review and network meta-analysis
Source: Ann Intensive Care. 2026 Aug 4;16:100130. doi: 10.1016/j.aicoj.2026.100130 (PMC13499409; doi:10.1016/j.aicoj.2026.100130)

Prebiotics, probiotics, and synbiotics therapy in critically ill patients: a systematic review and network meta-analysis

Supplemental Contents

eTable 1. Search strategies
eTable 2. Study-level event counts for gastrointestinal symptoms
eTable 3. The surface under the cumulative ranking curve
eTable 4. Pairwise meta-analysis for gastrointestinal symptoms
eTable 5. Global inconsistency
eTable 6. Local inconsistency
eTable 7. League table

eFigure 1. The risk of bias assessment for gastrointestinal symptoms
eFigure 2. The comparison-adjusted funnel plots for gastrointestinal symptoms
eFigure 3. The network map
eFigure 4. The forest plot
eFigure 5. The comparison-adjusted funnel plots
eFigure 6. The network map for subgroup analysis and sensitivity analysis of gastrointestinal symptoms
eFigure 7. The forest plot for subgroup analysis and sensitivity analysis of gastrointestinal symptoms
eFigure 8. The comparison-adjusted funnel plots for subgroup analysis and sensitivity analysis of gastrointestinal symptoms

eTable 1. Search strategies

a) PubMed search strategy (Performed on June 2, 2025)

	Search for	Results
#1	(((((Intensive Care Units[MeSH Terms]) OR (intensive care[Title/Abstract])) OR (Critical Illness[MeSH Terms])) OR (critical care[MeSH Terms])) OR (critically ill[Title/Abstract])) OR (critical care[Title/Abstract])	352,603
#2	((((((((((((((((((((prebiotics[MeSH Terms]) OR (prebiotics[Title/Abstract])) OR (dietary Fiber[MeSH Terms])) OR (oligosaccharides[MeSH Terms])) OR (oligofructose[Title/Abstract])) OR (xylooligosaccharide[Title/Abstract])) OR (galactooligosaccharide[Title/Abstract])) OR (lactosucrose[Title/Abstract])) OR (fructooligosaccharide[Title/Abstract])) OR (dextrins[MeSH Terms])) OR (polydextrose[Title/Abstract])) OR (chitin[Title/Abstract])) OR (Lignin[Title/Abstract])) OR (glucans[MeSH Terms])) OR (hemicellulose[Title/Abstract])) OR (cellulose[MeSH Terms])) OR (inulin[MeSH Terms])) OR (dietary supplements[MeSH Terms])) OR (polysaccharides[MeSH Terms])) OR (guar gum[Title/Abstract])) OR (((((((((((((((((((probiotics[MeSH Terms]) OR (probiotics[Title/Abstract])) OR (Lactobacillus[Title/Abstract])) OR (Bifidobacterium[Title/Abstract])) OR (Saccharomyces[Title/Abstract])) OR (enterococcus faecalis[Title/Abstract])) OR (enterococcus faecium[Title/Abstract])) OR (Escherichia Coli[Title/Abstract])) OR (bacillus cereus[Title/Abstract])) OR (bacillus clausii[Title/Abstract])) OR (bacillus coagulans[Title/Abstract])) OR (bacillus licheniformis[Title/Abstract])) OR (bacillus subtilis[Title/Abstract])) OR (Pediococcus[Title/Abstract])) OR (Leuconostoc[Title/Abstract])) OR (Akkermansia[Title/Abstract])) OR (Streptococcus[Title/Abstract])) OR (Clostridium butyricum[Title/Abstract])) OR (Faecalibacterium prausnitzii[Title/Abstract])) OR (next-generation probiotics[Title/Abstract]))) OR ((((synbiotics[MeSH Terms]) OR (synbiotics[Title/Abstract])) OR (pre[Title/Abstract] AND probiotics[Title/Abstract])) OR (prebiotics[Title/Abstract] AND probiotics[Title/Abstract]))	1,386,257
#3	((((((((randomized controlled trial[Publication Type]) OR (controlled clinical trial[Publication Type])) OR (randomized[Title/Abstract])) OR (placebo[Title/Abstract])) OR (drug therapy[MeSH Subheading])) OR (randomly[Title/Abstract])) OR (trial[Title/Abstract])) OR (groups[Title/Abstract])) NOT ((animals[MeSH Terms]) NOT (humans[MeSH Terms]))	5,616,700
#4	#1 AND #2 AND #3	3,726

b) CENTRAL search strategy (Performed on June 2, 2025)

#1	MeSH descriptor: [Intensive Care Units] explode all trees	6,210
#2	MeSH descriptor: [Critical Illness] explode all trees	3,773
#3	(critical illness):ti,ab,kw	6,614
#4	(critically ill):ti,ab,kw	9,502
#5	MeSH descriptor: [Critical Care] explode all trees	3,044
#6	("critical care"):ti,ab,kw	5,612
#7	(intensive care):ti,ab,kw	41,047
#8	#1 OR #2 OR #3 OR #4 OR #5 OR #6 OR #7	49,166
#9	MeSH descriptor: [Prebiotics] explode all trees	744
#10	(Prebiotics):ti,ab,kw	1,513
#11	MeSH descriptor: [Dietary Fiber] explode all trees	2,702
#12	MeSH descriptor: [Oligosaccharides] explode all trees	4,129
#13	(oligofructose):ti,ab,kw	211
#14	(xylooligosaccharide):ti,ab,kw	30
#15	(galactooligosaccharide):ti,ab,kw	110
#16	(lactosucrose):ti,ab,kw	7
#17	(fructooligosaccharide):ti,ab,kw	234
#18	MeSH descriptor: [Dextrins] explode all trees	400
#19	(polydextrose):ti,ab,kw	144
#20	(chitin):ti,ab,kw	114
#21	(Lignin):ti,ab,kw	47
#22	MeSH descriptor: [Glucans] explode all trees	4,972
#23	(hemicellulose):ti,ab,kw	32
#24	MeSH descriptor: [Cellulose] explode all trees	1,084
#25	MeSH descriptor: [Inulin] explode all trees	333

#26	MeSH descriptor: [Dietary Supplements] explode all trees	19,541
#27	MeSH descriptor: [Polysaccharides] explode all trees	21,343
#28	(guar gum):ti,ab,kw	290
#29	#9 OR #10 OR #11 OR #12 OR #13 OR #14 OR #15 OR #16 OR #17 OR #18 OR #19 OR #20 OR #21 OR #22 OR #23 OR #24 OR #25 OR #26 OR #27 OR #28	42,560
#30	MeSH descriptor: [Probiotics] explode all trees	3,647
#31	(probiotics):ti,ab,kw	8,618
#32	(Lactobacillus):ti,ab,kw	6,914
#33	(Bifidobacterium):ti,ab,kw	4,032
#34	(Saccharomyces):ti,ab,kw	701
#35	(enterococcus faecalis):ti,ab,kw	618
#36	(enterococcus faecium):ti,ab,kw	214
#37	(Escherichia coli):ti,ab,kw	3,419
#38	(bacillus cereus):ti,ab,kw	39
#39	(bacillus clausii):ti,ab,kw	97
#40	(bacillus coagulans):ti,ab,kw	223
#41	(bacillus licheniformis):ti,ab,kw	44
#42	(bacillus subtilis):ti,ab,kw	251
#43	(Pediococcus):ti,ab,kw	95
#44	(Leuconostoc):ti,ab,kw	41
#45	(Akkermansia):ti,ab,kw	240
#46	(Streptococcus):ti,ab,kw	5,929
#47	(Clostridium butyricum):ti,ab,kw	132
#48	(Faecalibacterium prausnitzii):ti,ab,kw	172
#49	(next-generation probiotics):ti,ab,kw	0
#50	#30 OR #31 OR #32 OR #33 OR #34 OR #35 OR #36 OR #37 OR #38 OR #39 OR	21,686

	#40 OR #41 OR #42 OR #43 OR #44 OR #45 OR #46 OR #47 OR #48 OR #49	
#51	MeSH descriptor: [Synbiotics] explode all trees	301
#52	(synbiotics):ti,ab,kw	742
#53	(pre and probitotics):ti,ab,kw	0
#54	(prebiotics and probiotics):ti,ab,kw	653
#55	#51 OR #52 OR #53 OR #54	1,195
#56	#29 OR #50 OR #55	59,162
#57	#8 AND #56	1,503
#58	#57 AND trials	1,468

c) Web of Science search strategy (Performed on June 2, 2025)

#1	TS=(Intensive Care Units) and Preprint Citation Index (Exclude – Database)	216,944
#2	TS=(intensive care) and Preprint Citation Index (Exclude – Database)	281,361
#3	TS=(Critical Illness) and Preprint Citation Index (Exclude – Database)	77,202
#4	TS=(critical care) and Preprint Citation Index (Exclude – Database)	231,234
#5	TS=(critically ill) and Preprint Citation Index (Exclude – Database)	89,538
#6	#1 OR #2 OR #3 OR #4 OR #5 and Preprint Citation Index (Exclude – Database)	495,877
#7	TS=(prebiotics) and Preprint Citation Index (Exclude – Database)	19,368
#8	TS=(dietary Fiber) and Preprint Citation Index (Exclude – Database)	67,446
#9	TS=(oligosaccharides) and Preprint Citation Index (Exclude – Database)	78,487
#10	TS=(oligofructose) and Preprint Citation Index (Exclude – Database)	2,303
#11	TS=(xylooligosaccharide) and Preprint Citation Index (Exclude – Database)	1,145
#12	TS=(galactooligosaccharide) and Preprint Citation Index (Exclude – Database)	883
#13	TS=(lactosucrose) and Preprint Citation Index (Exclude – Database)	144
#14	TS=(fructooligosaccharide) and Preprint Citation Index (Exclude – Database)	2,326
#15	TS=(dextrins) and Preprint Citation Index (Exclude – Database)	6,140

#16	TS=(polydextrose) and Preprint Citation Index (Exclude – Database)	818
#17	TS=(chitin) and Preprint Citation Index (Exclude – Database)	34,507
#18	TS=(Lignin) and Preprint Citation Index (Exclude – Database)	96,765
#19	TS=(glucans) and Preprint Citation Index (Exclude – Database)	21,492
#20	TS=(hemicellulose) and Preprint Citation Index (Exclude – Database)	30,760
#21	TS=(cellulose) and Preprint Citation Index (Exclude – Database)	230,414
#22	TS=(inulin) and Preprint Citation Index (Exclude – Database)	14,432
#23	TS=(dietary supplements) and Preprint Citation Index (Exclude – Database)	240,592
#24	TS=(polysaccharides) and Preprint Citation Index (Exclude – Database)	196,415
#25	TS=(guar gum) and Preprint Citation Index (Exclude – Database)	7,953
#26	#7 OR #8 OR #9 OR #10 OR #11 OR #12 OR #13 OR #14 OR #15 OR #16 OR #17 OR #18 OR #19 OR #20 OR #21 OR #22 OR #23 OR #24 OR #25 and Preprint Citation Index (Exclude – Database)	858,131
#27	TS=(probiotics) and Preprint Citation Index (Exclude – Database)	68,155
#28	TS=(Lactobacillus) and Preprint Citation Index (Exclude – Database)	98,973
#29	TS=(Bifidobacterium) and Preprint Citation Index (Exclude – Database)	27,819
#30	TS=(Saccharomyces) and Preprint Citation Index (Exclude – Database)	217,208
#31	TS=(enterococcus faecalis) and Preprint Citation Index (Exclude – Database)	29,665
#32	TS=(enterococcus faecium) and Preprint Citation Index (Exclude – Database)	17,400
#33	TS=(Escherichia Coli) and Preprint Citation Index (Exclude – Database)	681,890
#34	TS=(bacillus cereus) and Preprint Citation Index (Exclude – Database)	24,512
#35	TS=(bacillus clausii) and Preprint Citation Index (Exclude – Database)	481
#36	TS=(bacillus coagulans) and Preprint Citation Index (Exclude – Database)	2,094
#37	TS=(bacillus licheniformis) and Preprint Citation Index (Exclude – Database)	9,000
#38	TS=(bacillus subtilis) and Preprint Citation Index (Exclude – Database)	86,152
#39	TS=(Pediococcus) and Preprint Citation Index (Exclude – Database)	6,549

#40	TS=(Leuconostoc) and Preprint Citation Index (Exclude – Database)	7,660
#41	TS=(Akkermansia) and Preprint Citation Index (Exclude – Database)	6,692
#42	TS=(Streptococcus) and Preprint Citation Index (Exclude – Database)	151,350
#43	TS=(Clostridium butyricum) and Preprint Citation Index (Exclude – Database)	2,619
#44	TS=(Faecalibacterium prausnitzii) and Preprint Citation Index (Exclude – Database)	2,518
#45	TS=(next-generation probiotics) and Preprint Citation Index (Exclude – Database)	968
#46	#27 OR #28 OR #29 OR #30 OR #31 OR #32 OR #33 OR #34 OR #35 OR #36 OR #37 OR #38 OR #39 OR #40 OR #41 OR #42 OR #43 OR #44 OR #45 and Preprint Citation Index (Exclude – Database)	1,173,685
#47	TS=(synbiotics) and Preprint Citation Index (Exclude – Database)	3,771
#48	TS=(pre AND probiotics) and Preprint Citation Index (Exclude – Database)	2,926
#49	TS=(prebiotics AND probiotics) and Preprint Citation Index (Exclude – Database)	10,721
#50	#47 OR #48 OR #49 and Preprint Citation Index (Exclude – Database)	14,142
#51	#26 OR #46 OR #50 and Preprint Citation Index (Exclude – Database)	1,938,416
#52	TS=(randomized controlled trial) and Preprint Citation Index (Exclude – Database)	684,324
#53	TS=(controlled clinical trial) and Preprint Citation Index (Exclude – Database)	700,137
#54	TS=(randomly) and Preprint Citation Index (Exclude – Database)	561,115
#55	#52 OR #53 OR #54 and Preprint Citation Index (Exclude – Database)	1,411,318
#56	#55 AND #51 AND #6 and Preprint Citation Index (Exclude – Database)	1,781

eTable 2. Study-level event counts for gastrointestinal symptoms

Events and totals are reported as extracted from each trial for specified gastrointestinal outcomes and follow-ups. Asterisks in the control column indicate usual care controls that were explicitly confirmed to be fiber-free and included in the sensitivity analysis restricted to limited control group. *GI* gastrointestinal

Author	Outcome	Intervention	Total_I	Events_I	Control	Total_C	Events_C
Mahmoodpoor 2023	Diarrhea	probiotics	20	0	placebo	20	7
Johnstone 2021	Diarrhea	probiotics	1318	756	placebo	1332	731
Wongseree 2023	Diarrhea	synbiotics	12	1	usual care*	12	2
Prasoon 2022	Diarrhea	probiotics	52	0	usual care	50	4
Kooshki 2018	Diarrhea	prebiotics	30	0	usual care	30	4
Rohith 2023	Diarrhea	synbiotics	39	3	placebo	37	5
Morrow 2010	Diarrhea	probiotics	68	42	prebiotics	70	44
Chen 2021	Diarrhea	prebiotics	24	2	usual care*	22	9
Park 2025	Diarrhea	prebiotics	30	15	placebo	30	12
Gatt 2010	GI	synbiotics	25	4	placebo	25	6
Yagmurdur 2016	Diarrhea	prebiotics	60	22	usual care*	60	38
Tsilika 2022	Diarrhea	probiotics	59	0	placebo	53	2
Lu 2024	Constipation	probiotics	12	2	usual care	12	7
Dehghani 2023	Aspiration	synbiotics	44	3	usual care	48	8
Xi 2017	Diarrhea	prebiotics	62	7	usual care*	63	16
Barraud 2010	Diarrhea	probiotics	87	48	placebo	80	42
Wang 2021	Diarrhea	probiotics	28	12	placebo	33	8
Seifi 2022	Constipation	synbiotics	20	3	placebo	18	3
Majid 2014	Diarrhea	prebiotics	12	4	placebo	10	6
Shimizu 2018	Diarrhea	synbiotics	35	2	usual care	37	10
Mahmoodpoor 2019	Diarrhea	probiotics	48	7	placebo	54	15
Chittawatanarat 2010	Diarrhea	prebiotics	17	4	usual care*	17	8
Rushdi 2004	Vomiting	prebiotics	10	0	usual care*	10	2
Tuncay 2018	Diarrhea	prebiotics	23	2	usual care*	23	13
Knight 2009	Diarrhea	synbiotics	130	7	placebo	129	9
Dobb 1990	Diarrhea	prebiotics	45	16	usual care*	46	13
Schultz 2000	Diarrhea	prebiotics	22	5	placebo	22	7
Spapen 2001	Diarrhea	prebiotics	13	6	usual care*	12	11
Bleichner 1997	Diarrhea	probiotics	64	18	placebo	64	24
Naslowski 2025	Diarrhea	synbiotics	34	0	placebo	36	4
Kasiri 2023	Diarrhea	synbiotics	40	15	placebo	40	26
Rayes 2002	GI	synbiotics	31	6	prebiotics	32	11
Vahdat Shariatpanahi 2018	Diarrhea	prebiotics	16	1	placebo	16	3

eTable 3. The surface under the cumulative ranking curve

Surface under the cumulative ranking curve (SUCRA) values were calculated from the cumulative ranking probabilities estimated in the random effects NMA. SUCRA ranges from 0 to 1, with higher values indicating a greater probability of being among the best-ranked interventions (0 = worst, 1 = best). For dichotomous outcomes, rankings were based on the lowest relative risk being the best; for continuous outcomes (length of ICU stay and duration of mechanical ventilation), rankings were based on the shortest duration being the best. In the subgroup/sensitivity analyses, the SUCRA was computed using the evidence available within each restricted network, and the values were not estimable when an intervention was not represented in that network. The limited control group was defined as placebo or usual care explicitly confirmed to be fiber-free. SUCRA values for infectious complications are presented for reference only and were not interpreted because significant inconsistency was detected for this outcome.

GRV gastric residual volume, *ICU* intensive care unit, *NA* not applicable, *VAP* ventilator-associated pneumonia

Primary outcome and secondary outcomes

	Control	Prebiotics	Probiotics	Synbiotics
Gastrointestinal symptoms	0	0.7	0.3	0.9
Diarrhea	0.1	0.7	0.3	0.9
GRV/feeding intolerance	0.1	0.7	0.9	0.3
Mortality	0.1	0.8	0.4	0.7
ICU Mortality	0.2	0.7	0.3	0.8
Length of ICU stay	0	0.7	0.6	0.6
Mechanical ventilation duration	0.1	0.7	0.4	0.8
Infectious complications	0.1	0.3	0.8	0.8
VAP	0.1	0.3	0.7	0.8
Adverse events	0.6	0.6	0.2	0.6

Subgroup analysis of gastrointestinal symptoms

	Control	Prebiotics	Probiotics	Synbiotics
Patient population				
Internal medicine	0.3	0.9	0.1	0.7
Surgical	0.1	0.4	0.7	0.8
Duration of the intervention				
7 to 13 days	0.1	0.6	0.3	0.9
Oligosaccharides addition	0	0.8	NA	0.7

Sensitivity analysis of gastrointestinal symptoms

	Control	Prebiotics	Probiotics	Synbiotics

Exclude studies with high risk of bias	0.1	0.6	0.3	1.0
Excluding small studies (<50 participants)	0.1	0.5	0.3	1.0
Limited control group	0.1	0.7	0.3	0.9

eTable 4. Pairwise meta-analysis for gastrointestinal symptoms

Random-effects pairwise meta-analysis of direct (head-to-head) comparisons. Risk ratios <1 favored the first intervention. Random-effects models were fitted using restricted maximum likelihood. Statistical heterogeneity was quantified using I^2 and tested using Cochran's Q test (p for heterogeneity). For comparisons informed by a single trial, the I^2 and p for heterogeneity were not applicable. Control refers to placebo or usual care.

Comparison	Studies (n)	RR (95% CI)	I^2 (%)	p for heterogeneity
Prebiotics vs Control	13	0.58 (0.41–0.81)	46.9	0.048
Probiotics vs Control	9	0.88 (0.66–1.17)	53.7	0.03
Synbiotics vs Control	9	0.56 (0.40–0.78)	0.0	0.85
Probiotics vs Prebiotics	1	0.98 (0.76–1.27)	-	-
Synbiotics vs Prebiotics	1	0.56 (0.24–1.34)	-	-

RR risk ratio, *CI* confidence interval

eTable 5. Global inconsistency

Global inconsistencies were assessed by using a design-by-treatment interaction model. If the df was missing, it was not available as a source file.

GRV gastric residual volume, *ICU* intensive care unit, *NA* not applicable, *VAP* ventilator-associated pneumonia

Primary outcome and secondary outcomes

	df	Chi-square	p value
Gastrointestinal symptoms	2	1.51	0.47
Diarrhea	1	1.22	0.27
GRV/feeding intolerance	NA	NA	NA
Mortality	2	1.45	0.48
ICU Mortality	1	0.02	0.89
Length of ICU stay	2	2.18	0.34
Mechanical ventilation duration	2	0.31	0.86
Infectious complications	2	7.83	0.02
VAP	1	1.48	0.22
Adverse events	1	0	0.95

Subgroup analysis of gastrointestinal symptoms

	df	Chi-square	p value
Patient population			
Internal medicine	NA	NA	NA
Surgical	1	0.65	0.42
Duration of the intervention			
7 to 13 days	1	0.11	0.74

Sensitivity analysis of gastrointestinal symptoms

	df	Chi-square	p value
Exclude studies with high risk of bias	2	6.52	0.04
Excluding small studies (<50 participants)	2	0.47	0.79
Limited control group	2	2.55	0.28

eTable 6. Local inconsistency

GRV gastric residual volume, *ICU* intensive care unit, *RR* risk ratio, *SE* standard error

<u>Primary outcome and secondary outcomes</u>

Gastrointestinal symptoms

Comparison (treatment)	Direct estimate (SE)	Indirect estimate (SE)	Inconsistency (difference; SE)	p value
prebiotics vs control	-0.494 (0.152)	-0.089 (0.279)	-0.405 (0.306)	0.185
probiotics vs control	-0.139 (0.176)	-0.488 (0.331)	0.349 (0.363)	0.337
synbiotics vs control	-0.607 (0.220)	-1.027 (0.549)	0.420 (0.589)	0.476
probiotics vs prebiotics	-0.018 (0.295)	0.331 (0.211)	-0.349 (0.363)	0.337
synbiotics vs prebiotics	-0.574 (0.527)	-0.154 (0.265)	-0.420 (0.589)	0.476

Coefficients are expressed on the log scale (e.g., log RR). Comparisons are presented as "Treatment (second) vs Treatment (first)." p values indicate local inconsistency (difference between direct and indirect evidence).

Diarrhea

Comparison (treatment)	Direct estimate (SE)	Indirect estimate (SE)	Inconsistency (difference; SE)	p value
prebiotics vs control	-0.495 (0.160)	-0.080 (0.352)	-0.415 (0.376)	0.270
probiotics vs control	-0.098 (0.174)	-0.513 (0.346)	0.415 (0.376)	0.270
probiotics vs prebiotics	-0.018 (0.306)	0.397 (0.218)	-0.415 (0.376)	0.270

Coefficients are expressed on the log scale, e.g., log RR. p values indicate local inconsistency between direct and indirect evidence. Control refers to placebo or usual care. The synbiotics versus control comparison was not estimable because side-splitting could not be performed for this comparison.

GRV/feeding intolerance

This network is a star-shaped structure centered on control; because no closed loops existed between treatments, no comparisons involving both direct and indirect evidence were possible. Therefore, evaluation of local inconsistency via side-split analysis was not feasible, and inconsistency analysis was not applicable.

Mortality

Comparison (treatment)	Direct estimate (SE)	Indirect estimate (SE)	Inconsistency (difference; SE)	p value
prebiotics vs control	-0.268 (0.146)	-0.099 (0.272)	-0.169 (0.308)	0.582
probiotics vs control	-0.054 (0.053)	-0.482 (0.374)	0.428 (0.378)	0.257
synbiotics vs control	-0.199 (0.121)	0.045 (0.431)	-0.244 (0.448)	0.587
probiotics vs prebiotics	-0.194 (0.348)	0.234 (0.147)	-0.428 (0.378)	0.257
synbiotics vs prebiotics	0.254 (0.410)	0.010 (0.181)	0.244 (0.448)	0.587

Coefficients are expressed on the log scale (e.g., log RR). Comparisons are presented as “Treatment (second) vs Treatment (first).”

ICU mortality

Comparison (treatment)	Direct estimate (SE)	Indirect estimate (SE)	Inconsistency (difference; SE)	p value
prebiotics vs control	-0.242 (0.193)	-0.352 (0.803)	0.110 (0.826)	0.894
synbiotics vs control	-0.316 (0.185)	-0.206 (0.805)	-0.110 (0.826)	0.894
synbiotics vs prebiotics	0.036 (0.781)	-0.074 (0.268)	0.110 (0.826)	0.894

Coefficients are expressed on the log scale (e.g., log RR). p values indicate local inconsistency (difference between direct and indirect evidence). “Probiotics vs control” could not be computed the node-splitting.

Length of ICU stay

Comparison (treatment)	Direct estimate (SE)	Indirect estimate (SE)	Inconsistency (difference; SE)	p value
prebiotics vs control	-2.164 (0.884)	0.247 (1.809)	-2.411 (2.011)	0.230
probiotics vs control	-1.527 (0.653)	-1.507 (2.994)	-0.020 (3.063)	0.995

synbiotics vs control	-1.002 (0.872)	-4.630 (2.282)	3.628 (2.443)	0.137
probiotics vs prebiotics	0.200 (2.873)	0.180 (1.062)	0.020 (3.063)	0.995
synbiotics vs prebiotics	-2.510 (2.125)	1.118 (1.205)	-3.628 (2.443)	0.137

Estimates are expressed on the effect scale used for length of ICU stay (mean difference, days). p values indicate local inconsistency (difference between direct and indirect evidence). Comparisons are presented as "Treatment (second) vs Treatment (first)."

Mechanical ventilation duration

Comparison (treatment)	Direct estimate (SE)	Indirect estimate (SE)	Inconsistency (difference; SE)	p value
prebiotics vs control	-2.998 (2.218)	-1.206 (2.569)	-1.792 (3.367)	0.595
probiotics vs control	-0.903 (1.014)	-2.807 (3.815)	1.904 (3.953)	0.630
synbiotics vs control	-2.727 (1.510)	-3.364 (4.048)	0.638 (4.288)	0.882
probiotics vs prebiotics	-0.100 (3.276)	1.804 (2.212)	-1.904 (3.953)	0.630
synbiotics vs prebiotics	-1.000 (3.592)	-0.362 (2.342)	-0.638 (4.288)	0.882

Estimates are expressed on the effect scale for mechanical ventilation duration (mean difference, days). p values indicate local inconsistency (difference between direct and indirect evidence). Comparisons are presented as "Treatment (second) vs Treatment (first)."

Infectious complications

Comparison (treatment)	Direct estimate (SE)	Indirect estimate (SE)	Inconsistency (difference; SE)	p value
prebiotics vs control	-0.553 (0.251)	0.661 (0.353)	-1.214 (0.431)	0.005
probiotics vs control	-0.466 (0.118)	-1.401 (0.684)	0.940 (0.693)	0.175
synbiotics vs control	-0.419 (0.148)	-1.446 (0.470)	1.027 (0.491)	0.037
probiotics vs prebiotics	-1.150 (0.646)	-0.209 (0.253)	-0.940 (0.693)	0.175
synbiotics vs prebiotics	-1.056 (0.405)	-0.030 (0.277)	-1.027 (0.491)	0.037

Coefficients are expressed on the log scale (e.g., log RR). p values indicate local inconsistency (difference between direct and indirect evidence). Comparisons are presented as “Treatment (second) vs Treatment (first).”

Ventilator-associated pneumonia (VAP)

Comparison (treatment)	Direct estimate (SE)	Indirect estimate (SE)	Inconsistency (difference; SE)	p value
prebiotics vs control	-0.504 (0.423)	0.255 (0.459)	-0.759 (0.625)	0.224
probiotics vs control	-0.408 (0.149)	-1.167 (0.606)	0.759 (0.625)	0.224
probiotics vs prebiotics	-0.663 (0.434)	0.096 (0.450)	-0.759 (0.625)	0.224

Coefficients are expressed on the log scale (e.g., log RR). p values indicate local inconsistency (difference between direct and indirect evidence). Comparisons are presented as “Treatment (second) vs Treatment (first).” Synbiotics-related node-splitting comparisons could not be computed.

Adverse events

Comparison (treatment)	Direct estimate (SE)	Indirect estimate (SE)	Inconsistency (difference; SE)	p value
prebiotics vs control	-0.050 (0.703)	0.066 (1.857)	-0.116 (1.986)	0.953
synbiotics vs control	0.018 (1.179)	-0.098 (1.598)	0.116 (1.986)	0.953
synbiotics vs prebiotics	-0.048 (1.435)	0.068 (1.373)	-0.116 (1.986)	0.953

Coefficients are expressed on the log scale (e.g., log RR). p values indicate local inconsistency (difference between direct and indirect evidence). Comparisons are presented as “Treatment (second) vs Treatment (first).” Probiotics vs control could not be computed in node-splitting.

<u>Subgroup analysis of gastrointestinal symptoms</u>

Patient population: Internal medicine

This network is a star-shaped structure centered on control; because no closed loops existed between treatments, no comparisons involving both direct and indirect evidence were possible. Therefore, evaluation of local inconsistency via side-split analysis was not feasible, and inconsistency analysis was not applicable.

Patient population: Surgical

Comparison (treatment)	Direct estimate (SE)	Indirect estimate (SE)	Inconsistency (difference; SE)	p value
prebiotics vs control	-0.693 (0.507)	-0.050 (0.616)	-0.643 (0.798)	0.420
synbiotics vs control	-0.624 (0.431)	-1.268 (0.672)	0.643 (0.798)	0.420
synbiotics vs prebiotics	-0.574 (0.441)	0.069 (0.666)	-0.643 (0.798)	0.420

Coefficients are expressed on the log scale (e.g., log RR). p values indicate local inconsistency (difference between direct and indirect evidence). Comparisons are presented as “Treatment (second) vs Treatment (first).” Probiotics vs control could not be computed in node-splitting.

Duration of the intervention: 7-13 days

Comparison (treatment)	Direct estimate (SE)	Indirect estimate (SE)	Inconsistency (difference; SE)	p value
prebiotics vs control	-0.392 (0.239)	-0.147 (0.701)	-0.244 (0.740)	0.741
synbiotics vs control	-0.722 (0.461)	-0.966 (0.580)	0.244 (0.740)	0.741
synbiotics vs prebiotics	-0.574 (0.528)	-0.330 (0.519)	-0.244 (0.740)	0.741

Coefficients are expressed on the log scale (e.g., log RR). p values indicate local inconsistency (difference between direct and indirect evidence). Comparisons are presented as “Treatment (second) vs Treatment (first).” Probiotics vs control could not be computed in node-splitting.

Oligosaccharides addition

This network is a star-shaped structure centered on control; because no closed loops existed between treatments, no comparisons involving both direct and indirect evidence were possible. Therefore, evaluation of local inconsistency via side-split analysis was not feasible, and inconsistency analysis was not applicable.

<u>Sensitivity Analysis of Gastrointestinal Symptoms</u>

High-Risk Bias Study Exclusion

Comparison (treatment)	Direct estimate (SE)	Indirect estimate (SE)	Inconsistency (difference; SE)	p value
prebiotics vs control	-0.443 (0.142)	0.051 (0.131)	-0.493 (0.193)	0.011
probiotics vs control	0.036 (0.033)	-0.422 (0.190)	0.459 (0.193)	0.017
synbiotics vs control	-0.569 (0.205)	-0.914 (0.522)	0.345 (0.559)	0.538
probiotics vs prebiotics	-0.018 (0.132)	0.441 (0.140)	-0.459 (0.193)	0.017
synbiotics vs prebiotics	-0.574 (0.492)	-0.230 (0.265)	-0.345 (0.559)	0.538

Coefficients are expressed on the log scale (e.g., log RR). p values indicate local inconsistency (difference between direct and indirect evidence). Comparisons are presented as 'Treatment (second) vs Treatment (first)'.

Excluding small studies (<50 participants)

Comparison (treatment)	Direct estimate (SE)	Indirect estimate (SE)	Inconsistency (difference; SE)	p value
prebiotics vs control	-0.250 (0.165)	-0.019 (0.232)	-0.231 (0.289)	0.424
probiotics vs control	-0.038 (0.138)	-0.247 (0.284)	0.209 (0.319)	0.511
synbiotics vs control	-0.627 (0.209)	-0.756 (0.505)	0.129 (0.545)	0.813
probiotics vs prebiotics	-0.018 (0.233)	0.192 (0.217)	-0.209 (0.319)	0.511
synbiotics vs prebiotics	-0.574 (0.482)	-0.445 (0.253)	-0.129 (0.545)	0.813

Notes: Coefficients are expressed on the log scale, e.g., log risk ratio. p values indicate local inconsistency, defined as the difference between direct and indirect evidence. Comparisons are presented as “Treatment second vs Treatment first”.

Limited control group

Comparison (treatment)	Direct estimate (SE)	Indirect estimate (SE)	Inconsistency (difference; SE)	p value
prebiotics vs control	-0.459 (0.138)	0.001 (0.230)	-0.460 (0.261)	0.078
probiotics vs control	-0.048 (0.145)	-0.451 (0.280)	0.403 (0.308)	0.190
synbiotics vs control	-0.486 (0.232)	-0.985 (0.530)	0.498 (0.579)	0.390
probiotics vs prebiotics	-0.018 (0.245)	0.386 (0.187)	-0.403 (0.308)	0.190
synbiotics vs prebiotics	-0.574 (0.510)	-0.076 (0.275)	-0.498 (0.579)	0.390

Coefficients are expressed on the log scale, e.g., log RR. p values indicate local inconsistency, defined as the difference between direct and indirect evidence. Comparisons are presented as "Treatment second vs Treatment first." Control refers to placebo or usual care.

eTable 7. League table

League tables present the pooled pairwise treatment effects (95% confidence intervals) from the random effects network meta-analysis comparing the control, prebiotic, probiotic, and synbiotic groups. Each cell reports the effect estimate for the intervention in the column, relative to the intervention in the row. For binary outcomes, estimates are expressed as risk ratios (RR); values <1 indicate a lower risk (i.e., favor the column-defining intervention). For continuous outcomes, estimates are presented as the mean difference (MD) in days; values <0 indicate a shorter duration (i.e., favoring the column-defining intervention). The diagonal entries indicate self-comparisons. Estimates for infectious complications are presented for reference only. Because significant global inconsistency (P = 0.02) and local inconsistency were detected for prebiotics versus control (P = 0.005) and synbiotics versus control (P = 0.037), the network meta-analysis estimates and treatment rankings for this outcome were not interpreted. The direct pairwise estimates reported in the main text should therefore be used.

Subgroup and sensitivity analyses.

GRV gastric residual volume, *ICU* intensive care unit, *VAP* ventilator-associated pneumonia.

Secondary outcomes

Diarrhea

	control	prebiotics	probiotics	synbiotics
control	-	0.65 (0.48, 0.88)	0.84 (0.61, 1.14)	0.52 (0.31, 0.87)
prebiotics	1.55 (1.14, 2.10)	-	1.30 (0.90, 1.86)	0.80 (0.45, 1.44)
probiotics	1.19 (0.88, 1.63)	0.77 (0.54, 1.11)	-	0.62 (0.34, 1.12)
synbiotics	1.92 (1.15, 3.22)	1.24 (0.69, 2.23)	1.61 (0.89, 2.90)	-

GRV/feeding intolerance

	control	prebiotics	probiotics	synbiotics
control	-	0.61 (0.48, 0.79)	0.51 (0.31, 0.84)	0.84 (0.51, 1.40)
prebiotics	1.63 (1.27, 2.09)	-	0.83 (0.48, 1.44)	1.37 (0.78, 2.41)
probiotics	1.97 (1.20, 3.24)	1.21 (0.69, 2.10)	-	1.66 (0.82, 3.37)
synbiotics	1.19 (0.72, 1.97)	0.73 (0.41, 1.28)	0.60 (0.30, 1.22)	-

Mortality

	control	prebiotics	probiotics	synbiotics
control	-	0.79 (0.62, 1.02)	0.94 (0.85, 1.04)	0.83 (0.66, 1.05)
prebiotics	1.26 (0.98, 1.62)	-	1.18 (0.91, 1.54)	1.05 (0.76, 1.45)
probiotics	1.06 (0.96, 1.18)	0.84 (0.65, 1.10)	-	0.89 (0.69, 1.14)
synbiotics	1.20 (0.95, 1.51)	0.95 (0.69, 1.32)	1.13 (0.88, 1.45)	-

ICU Mortality

	control	prebiotics	probiotics	synbiotics
control	-	0.78 (0.54, 1.13)	0.97 (0.85, 1.10)	0.73 (0.51, 1.04)
prebiotics	1.28 (0.89, 1.85)	-	1.24 (0.84, 1.83)	0.94 (0.57, 1.54)
probiotics	1.03 (0.91, 1.17)	0.80 (0.54, 1.19)	-	0.76 (0.52, 1.10)
synbiotics	1.36 (0.96, 1.94)	1.06 (0.65, 1.75)	1.32 (0.91, 1.93)	-

Length of ICU stay

	control	prebiotics	probiotics	synbiotics
control	-	-1.70 (-3.27, -0.14)	-1.52 (-2.75, -0.29)	-1.48 (-3.11, 0.16)
prebiotics	1.70 (0.14, 3.27)	-	0.19 (-1.73, 2.11)	0.23 (-1.86, 2.32)
probiotics	1.52 (0.29, 2.75)	-0.19 (-2.11, 1.73)	-	0.04 (-1.98, 2.06)
synbiotics	1.48 (-0.16, 3.11)	-0.23 (-2.32, 1.86)	-0.04 (-2.06, 1.98)	-

Mechanical ventilation duration

	control	prebiotics	Probiotics	synbiotics
control	-	-2.18 (-5.39, 1.02)	-1.03 (-2.91, 0.86)	-2.73 (-5.43, -0.03)
prebiotics	2.18 (-1.02, 5.39)	-	1.16 (-2.28, 4.60)	-0.54 (-4.25, 3.17)
probiotics	1.03 (-0.86, 2.91)	-1.16 (-4.60, 2.28)	-	-1.70 (-4.93, 1.53)
synbiotics	2.73 (0.03, 5.43)	0.54 (-3.17, 4.25)	1.70 (-1.53, 4.93)	-

Infectious complications

	control	prebiotics	probiotics	synbiotics
control	-	0.85 (0.56, 1.30)	0.61 (0.48, 0.77)	0.59 (0.44, 0.80)
prebiotics	1.17 (0.77, 1.79)	-	0.72 (0.45, 1.14)	0.70 (0.44, 1.11)
probiotics	1.64 (1.30, 2.07)	1.40 (0.88, 2.22)	-	0.97 (0.68, 1.39)
synbiotics	1.68 (1.26, 2.26)	1.44 (0.90, 2.28)	1.03 (0.72, 1.47)	-

VAP

	control	prebiotics	Probiotics	synbiotics
control	-	0.85 (0.45, 1.60)	0.64 (0.47, 0.85)	0.59 (0.37, 0.94)
prebiotics	1.17 (0.63, 2.20)	-	0.75 (0.40, 1.40)	0.70 (0.32, 1.51)
probiotics	1.57 (1.18, 2.11)	1.34 (0.72, 2.51)	-	0.93 (0.55, 1.60)
synbiotics	1.69 (1.07, 2.67)	1.44 (0.66, 3.11)	1.07 (0.63, 1.83)	-

Adverse events

	control	prebiotics	probiotics	synbiotics
control	-	0.96 (0.28, 3.32)	1.69 (0.57, 4.96)	0.98 (0.16, 6.15)
prebiotics	1.04 (0.30, 3.58)	-	1.75 (0.34, 9.03)	1.01 (0.15, 6.91)
probiotics	0.59 (0.20, 1.74)	0.57 (0.11, 2.94)	-	0.58 (0.07, 4.88)

synbiotics	1.02 (0.16, 6.45)	0.99 (0.14, 6.72)	1.73 (0.20, 14.56)	-

Subgroup Analysis of Gastrointestinal Symptoms

Internal medicine patients

	control	prebiotics	probiotics	synbiotics
control	-	0.54 (0.30, 0.94)	1.29 (0.63, 2.63)	0.66 (0.23, 1.90)
prebiotics	1.87 (1.06, 3.28)	-	2.40 (0.94, 6.10)	1.23 (0.37, 4.07)
probiotics	0.78 (0.38, 1.59)	0.42 (0.16, 1.06)	-	0.51 (0.14, 1.84)
synbiotics	1.51 (0.53, 4.33)	0.81 (0.25, 2.67)	1.94 (0.54, 6.95)	-

Surgical patients

	control	prebiotics	probiotics	synbiotics
control	-	0.65 (0.30, 1.40)	0.45 (0.21, 0.97)	0.44 (0.22, 0.90)
prebiotics	1.54 (0.72, 3.32)	-	0.69 (0.23, 2.06)	0.68 (0.33, 1.41)
probiotics	2.23 (1.03, 4.86)	1.45 (0.49, 4.32)	-	0.99 (0.35, 2.84)
synbiotics	2.25 (1.11, 4.59)	1.46 (0.71, 3.00)	1.01 (0.35, 2.89)	-

Intervention period: 7–13 days

	control	prebiotics	probiotics	synbiotics
control	-	0.70 (0.46, 1.07)	0.88 (0.55, 1.41)	0.44 (0.22, 0.88)
prebiotics	1.43 (0.94, 2.19)	-	1.27 (0.69, 2.32)	0.63 (0.31, 1.29)
probiotics	1.13 (0.71, 1.81)	0.79 (0.43, 1.44)	-	0.50 (0.22, 1.15)
synbiotics	2.26 (1.13, 4.51)	1.57 (0.78, 3.20)	2.00 (0.87, 4.57)	-

Oligosaccharides addition

	control	prebiotics	synbiotics
control	-	0.52 (0.38, 0.72)	0.53 (0.37, 0.77)
prebiotics	1.92 (1.39, 2.65)	-	1.02 (0.63, 1.67)
synbiotics	1.88 (1.30, 2.72)	0.98 (0.60, 1.60)	-

Sensitivity Analysis of Gastrointestinal Symptoms

High-Risk Bias Study Exclusion

	control	prebiotics	probiotics	synbiotics
control	-	0.74 (0.54, 1.00)	0.90 (0.70, 1.15)	0.54 (0.38, 0.78)
prebiotics	1.35 (1.00, 1.84)	-	1.22 (0.89, 1.67)	0.73 (0.47, 1.14)
probiotics	1.11 (0.87, 1.43)	0.82 (0.60, 1.13)	-	0.60 (0.39, 0.93)
synbiotics	1.85 (1.28, 2.67)	1.36 (0.87, 2.12)	1.66 (1.08, 2.55)	-

Excluding small studies (<50 participants)

	control	prebiotics	probiotics	synbiotics
control	-	0.84 (0.64, 1.10)	0.93 (0.74, 1.18)	0.53 (0.36, 0.76)
prebiotics	1.19 (0.91, 1.55)	-	1.11 (0.83, 1.49)	0.62 (0.41, 0.96)
probiotics	1.07 (0.85, 1.35)	0.90 (0.67, 1.21)	-	0.56 (0.37, 0.86)
synbiotics	1.90 (1.31, 2.76)	1.60 (1.04, 2.47)	1.78 (1.16, 2.72)	-

Limited control group

	control	prebiotics	probiotics	synbiotics
control	-	0.69 (0.52,0.90)	0.88 (0.67,1.14)	0.57 (0.38,0.85)
prebiotics	1.46 (1.11,1.91)	-	1.28 (0.93,1.75)	0.83 (0.52,1.31)
probiotics	1.14 (0.87,1.49)	0.78 (0.57,1.08)	-	0.65 (0.40,1.05)
synbiotics	1.76 (1.17,2.65)	1.21 (0.76,1.92)	1.54 (0.95,2.49)	-

eFigure 1. The risk of bias assessment for gastrointestinal symptoms

Risk of bias assessment was performed using the Cochrane Risk of Bias 2 (RoB 2) tool for the 33 studies included in the network meta-analysis for gastrointestinal symptoms. Trials reporting only continuous measures (e.g., gastric residual volume in mL) without dichotomous event data were not included.

Ctrl control, *Pre* prebiotics, *Pro* probiotics, *Syn* synbiotics

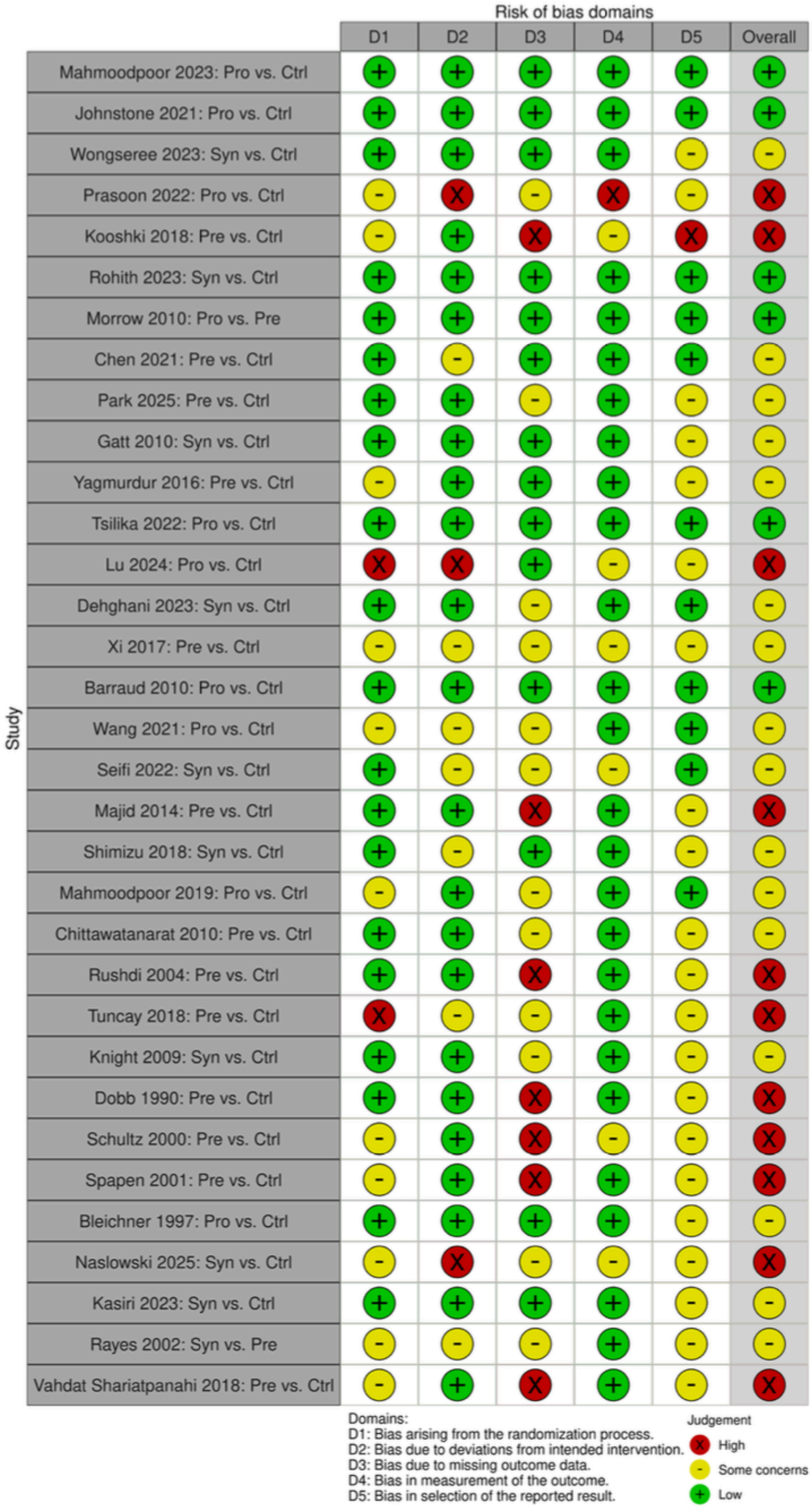

eFigure 2. Comparison-adjusted funnel plots for gastrointestinal symptoms

Comparison-adjusted funnel plot assessing the small-study effects of gastrointestinal symptoms. Each point represents a study with different colors, indicating different treatment comparisons. The x-axis shows the comparison-adjusted effect size (ES_adj), and the y-axis indicates the standard error.

Ctrl control, *Pre* prebiotics, *Pro* probiotics, *Syn* synbiotics

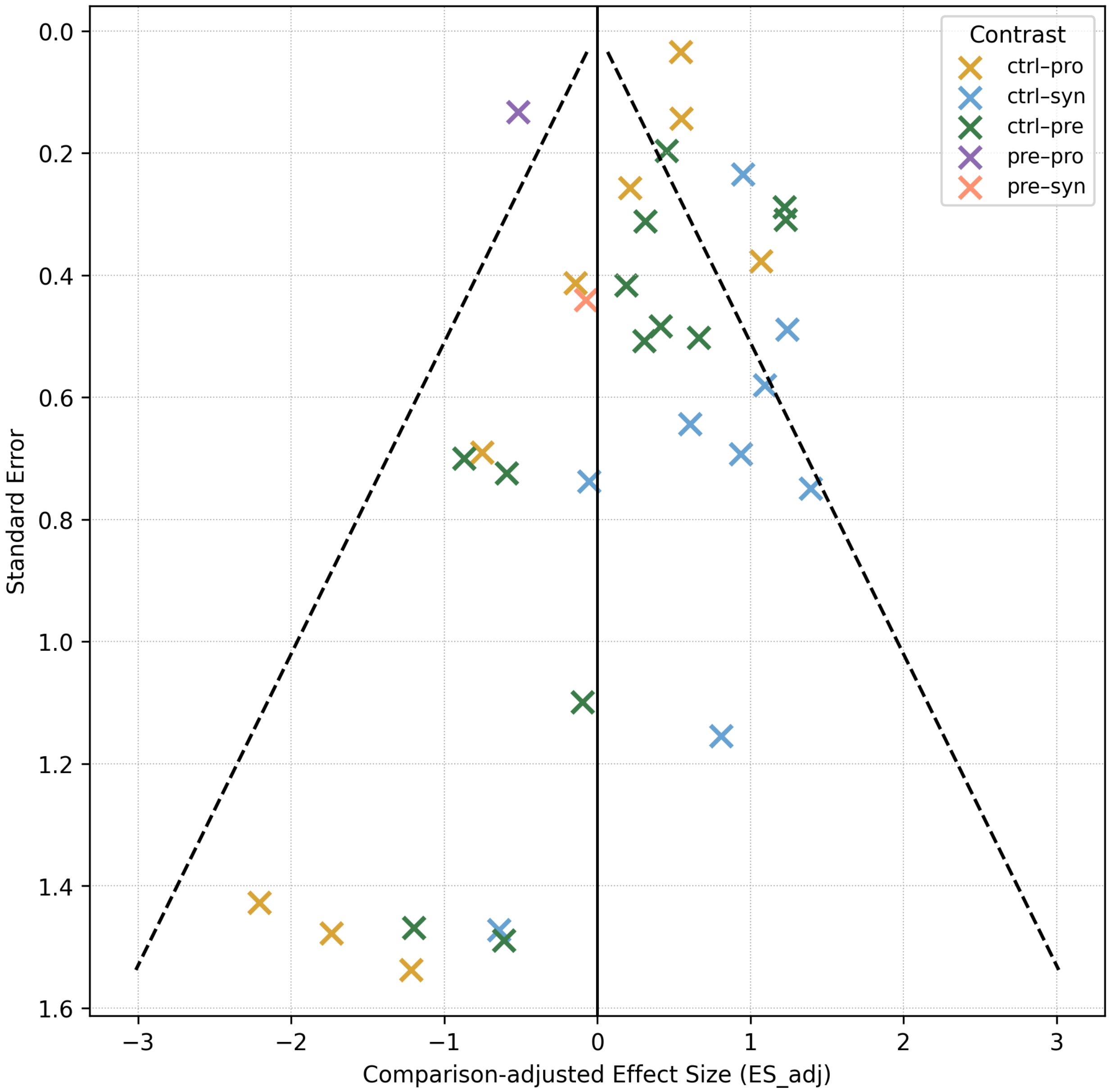

eFigure 3. The network map

Network plots showing the map of treatment comparisons for secondary outcomes: diarrhea, GRV/feeding intolerance, mortality, ICU mortality, length of ICU stay, duration of mechanical ventilation, infectious complications, VAP, and adverse events. The node size is proportional to the number of patients randomized to each intervention, and the edge thickness is proportional to the number of studies for each comparison.

GRV gastric residual volume, *ICU* intensive care unit, *VAP* ventilator-associated pneumonia

Diarrhea

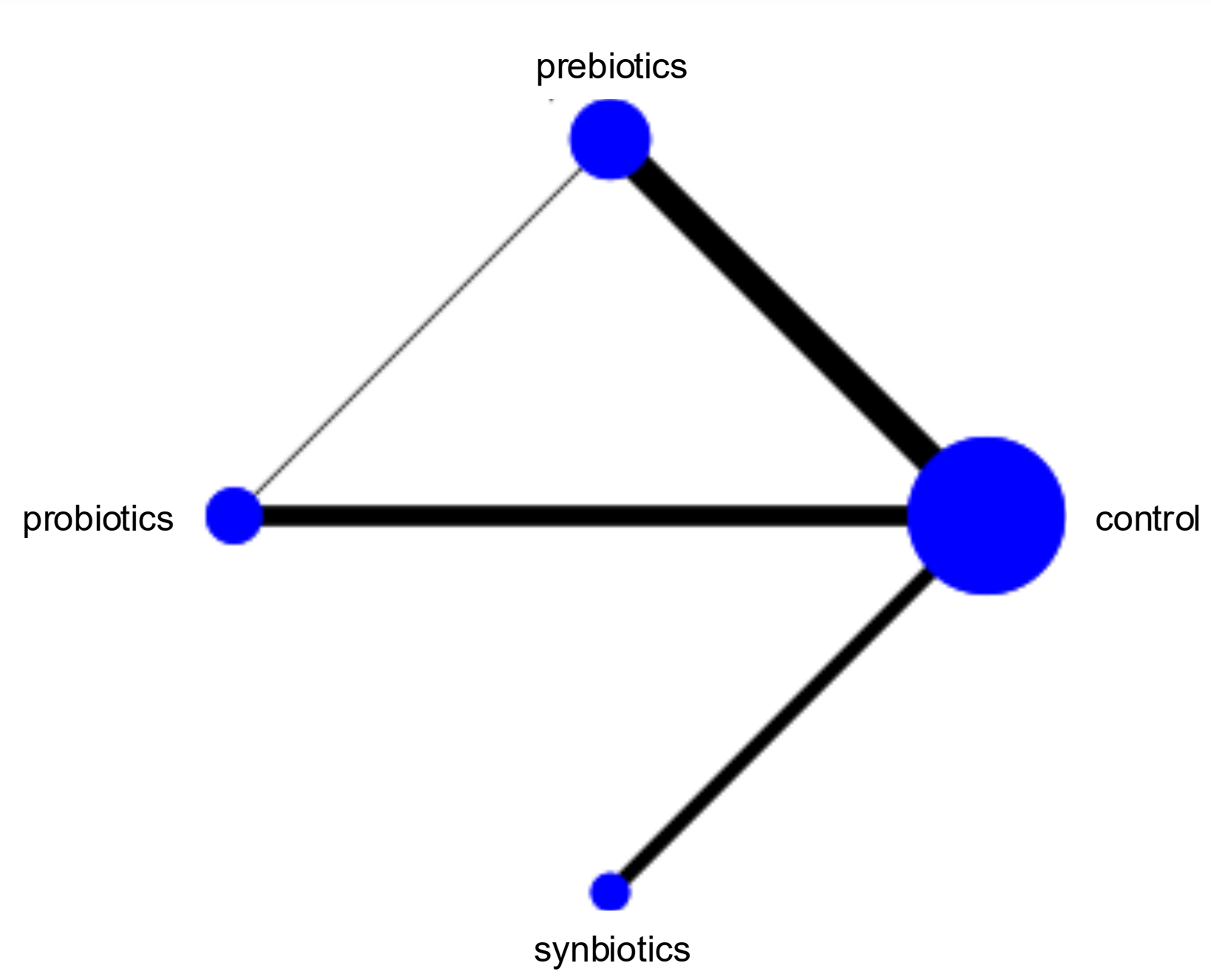

GRV/feeding intolerance

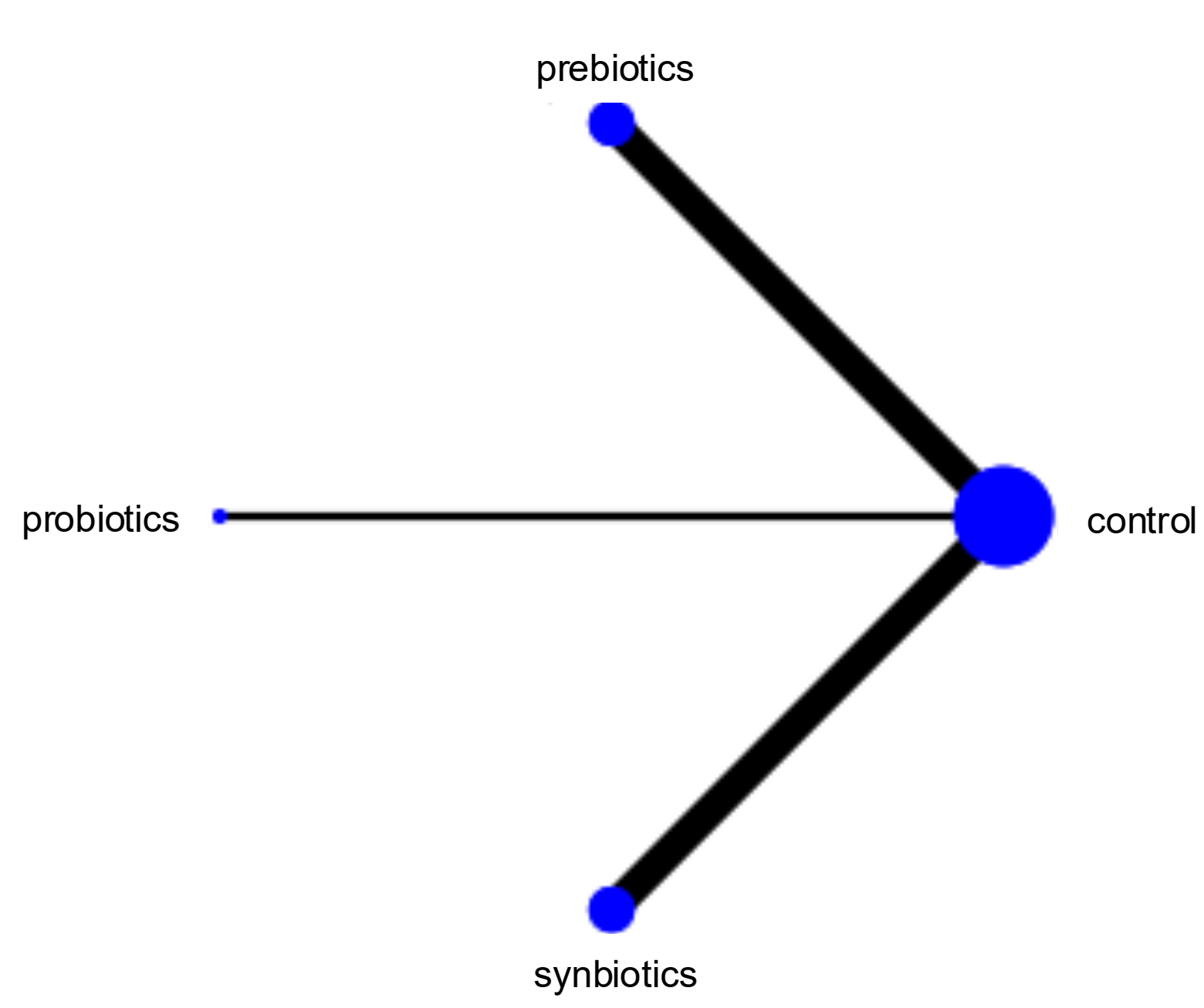

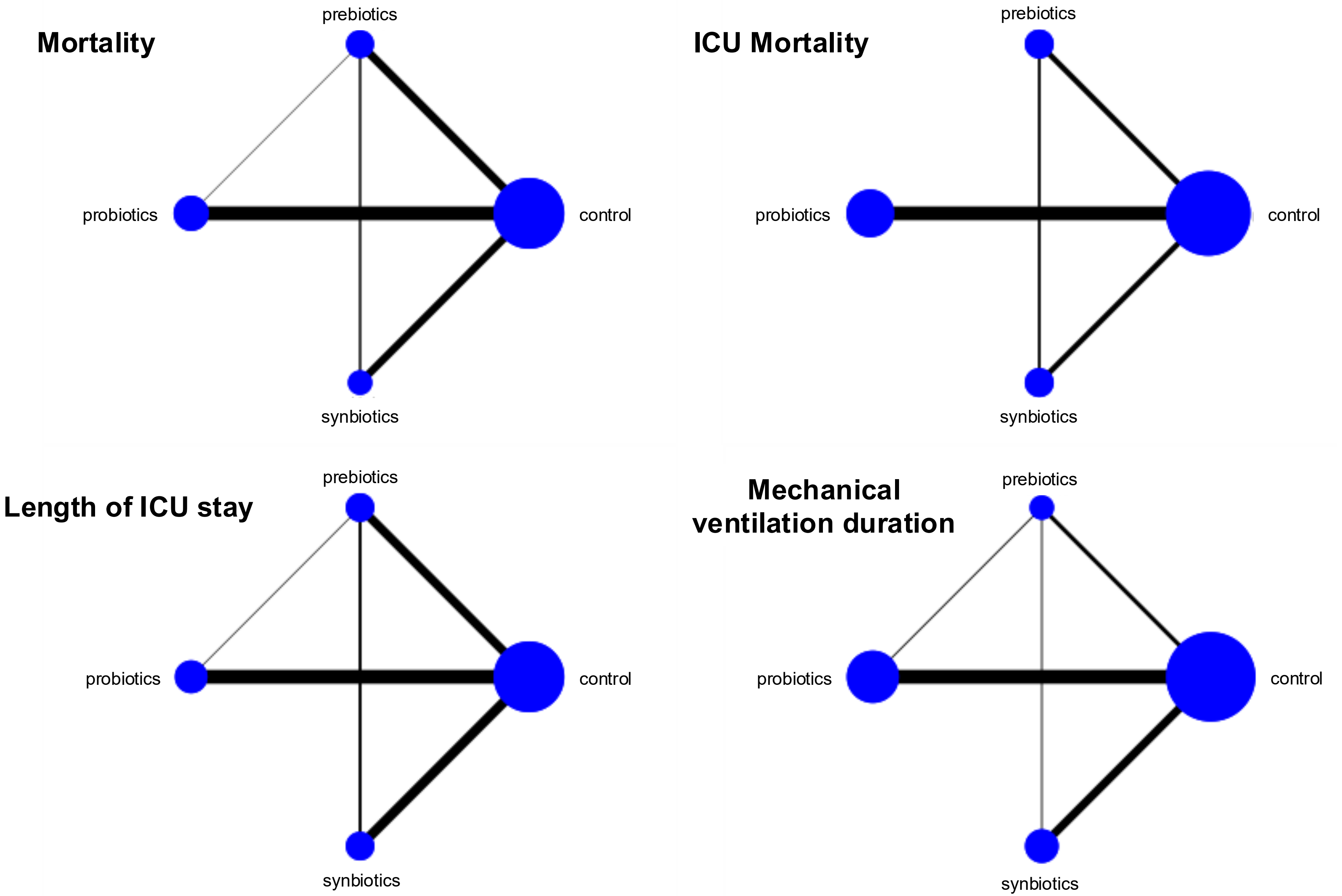
Mortality
prebiotics
probiotics
control
synbiotics
ICU Mortality
prebiotics
probiotics
control
synbiotics
Length of ICU stay
prebiotics
probiotics
control
synbiotics
Mechanical ventilation duration
prebiotics
probiotics
control
synbiotics

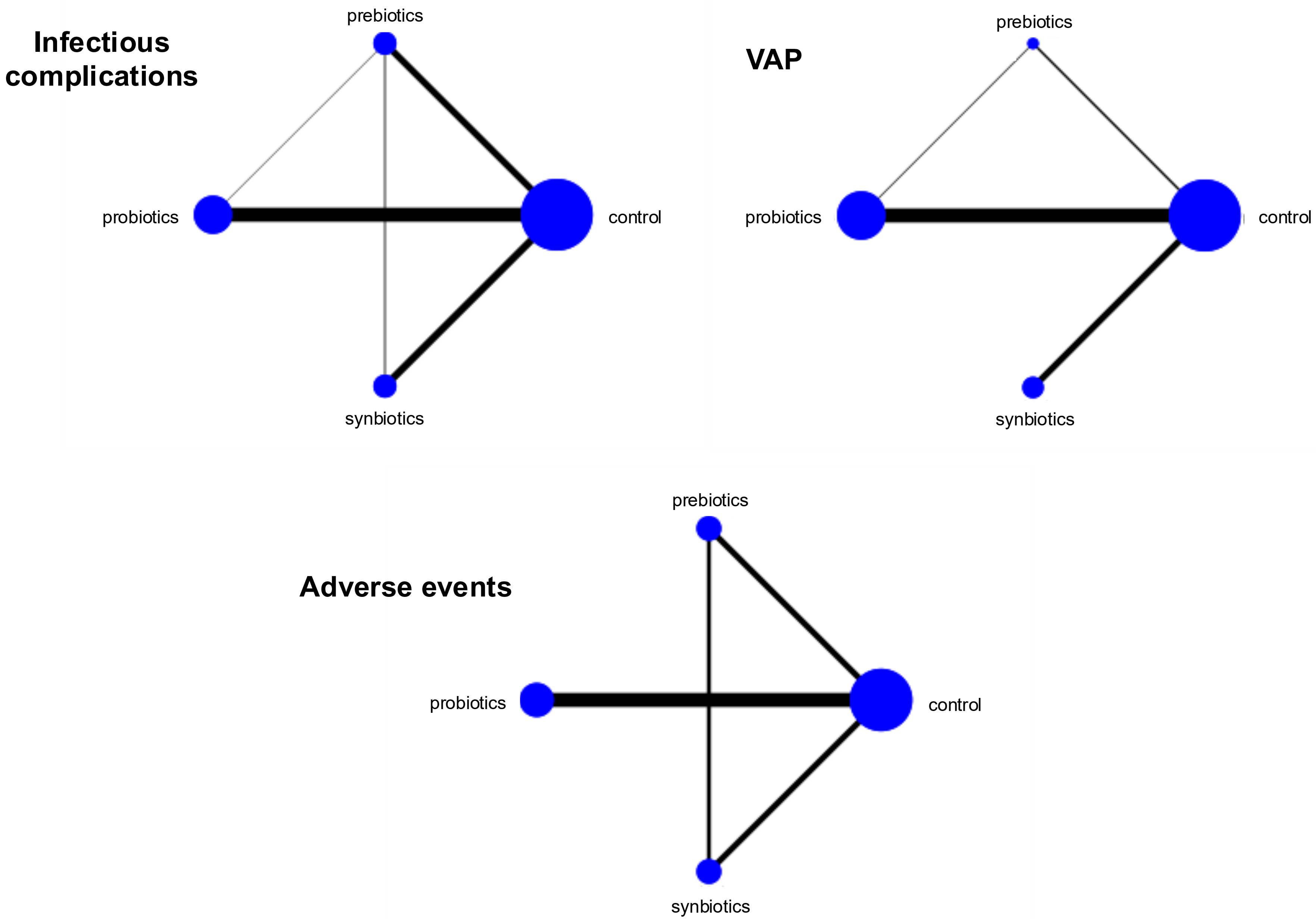
Infectious complications
prebiotics
probiotics
control
synbiotics
VAP
prebiotics
probiotics
control
synbiotics
Adverse events
prebiotics
probiotics
control
synbiotics

eFigure 4. The forest plot

Forest plots demonstrating the results of the network meta-analysis for secondary outcomes compared with controls: diarrhea, GRV/feeding intolerance, mortality, ICU mortality, length of ICU stay, duration of mechanical ventilation, infectious complications, VAP, and adverse events. For binary outcomes, the results are expressed as risk ratios with 95% confidence intervals; values <1 favor active intervention. Regarding continuous outcomes (length of ICU stay and duration of mechanical ventilation), results are expressed as mean differences in days; values <0 favor active intervention. Estimates for infectious complications are presented for reference only. Because significant global inconsistency (P = 0.02) and local inconsistency were detected for prebiotics versus control (P = 0.005) and synbiotics versus control (P = 0.037), the network meta-analysis estimates and treatment rankings for this outcome were not interpreted. The direct pairwise estimates reported in the main text should therefore be used.

GRV gastric residual volume, *ICU* intensive care unit, *VAP* ventilator-associated pneumonia

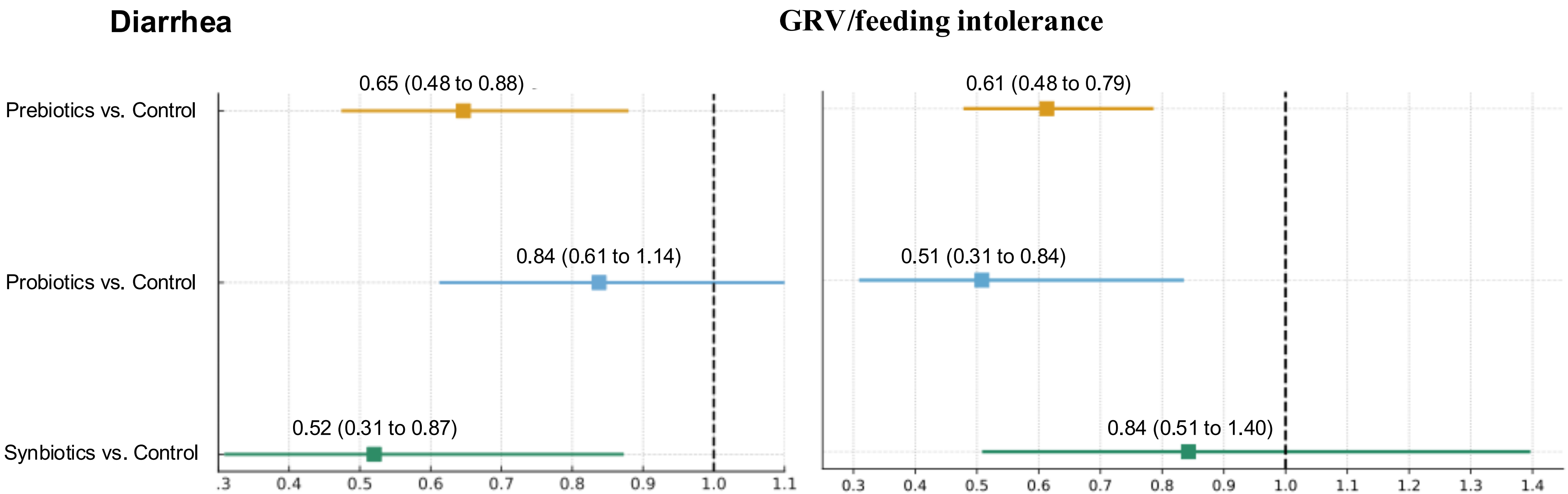

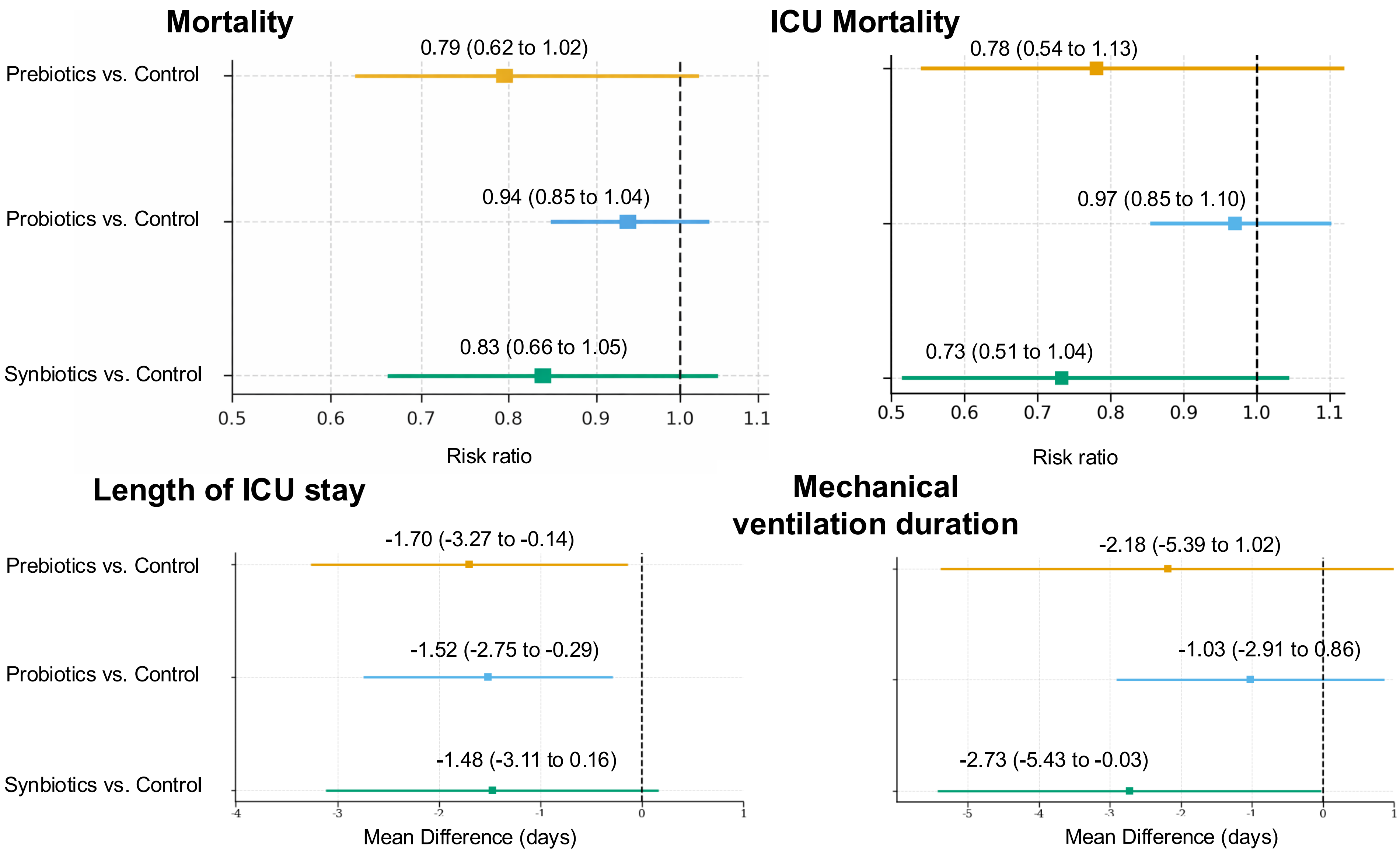
Mortality
0.79 (0.62 to 1.02)
Prebiotics vs. Control
0.94 (0.85 to 1.04)
Probiotics vs. Control
0.83 (0.66 to 1.05)
Synbiotics vs. Control
0.5
0.6
0.7
0.8
0.9
1.0
1.1
Risk ratio
ICU Mortality
0.78 (0.54 to 1.13)
0.97 (0.85 to 1.10)
0.73 (0.51 to 1.04)
0.5
0.6
0.7
0.8
0.9
1.0
1.1
Risk ratio
Length of ICU stay
-1.70 (-3.27 to -0.14)
Prebiotics vs. Control
-1.52 (-2.75 to -0.29)
Probiotics vs. Control
-1.48 (-3.11 to 0.16)
Synbiotics vs. Control
Mean Difference (days)
Mechanical ventilation duration
-2.18 (-5.39 to 1.02)
-1.03 (-2.91 to 0.86)
-2.73 (-5.43 to -0.03)
Mean Difference (days)

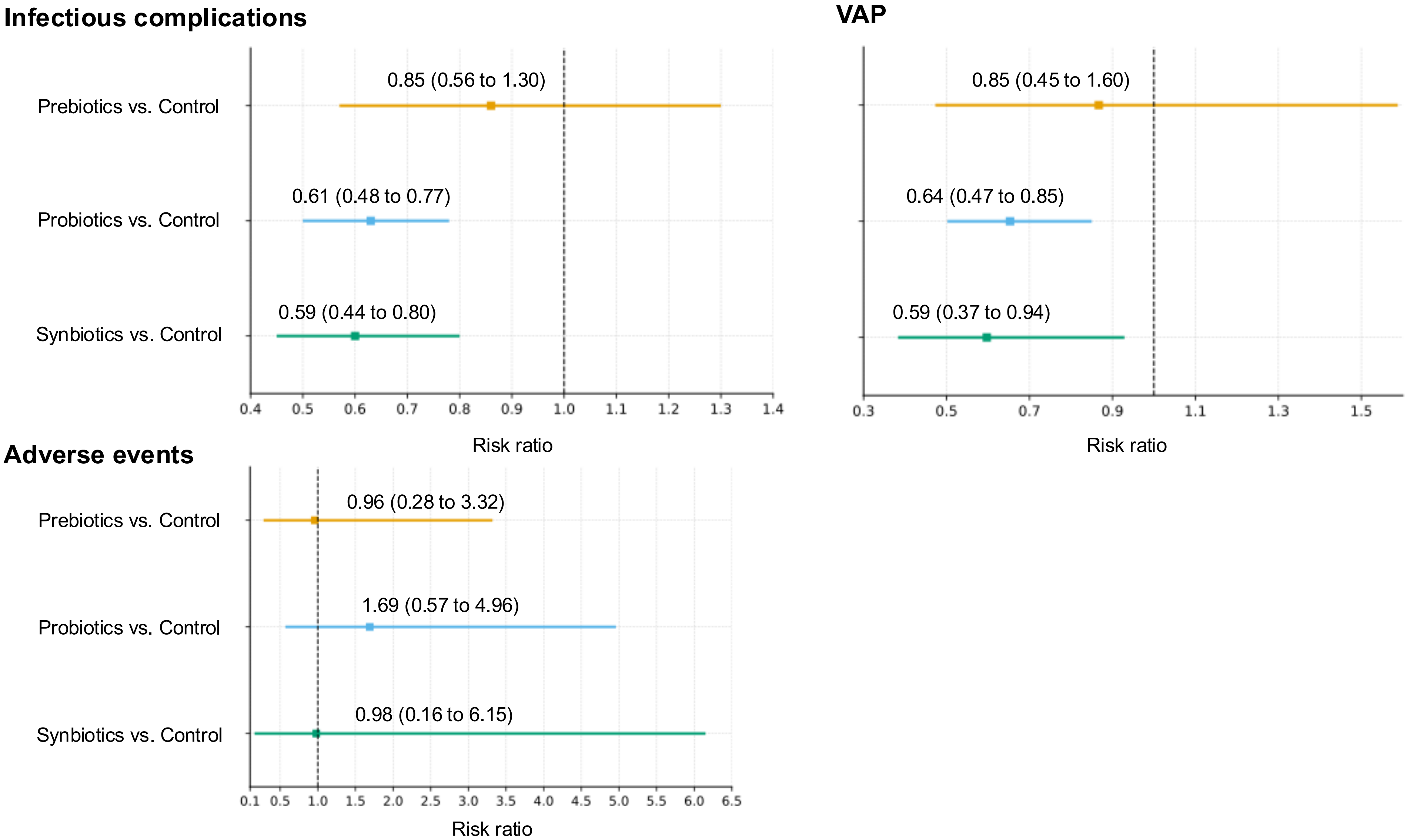
Infectious complications
0.85 (0.56 to 1.30)
Prebiotics vs. Control
0.61 (0.48 to 0.77)
Probiotics vs. Control
0.59 (0.44 to 0.80)
Synbiotics vs. Control
0.4 0.5 0.6 0.7 0.8 0.9 1.0 1.1 1.2 1.3 1.4
Risk ratio
VAP
0.85 (0.45 to 1.60)
0.64 (0.47 to 0.85)
0.59 (0.37 to 0.94)
0.3 0.5 0.7 0.9 1.1 1.3 1.5
Risk ratio
Adverse events
0.96 (0.28 to 3.32)
Prebiotics vs. Control
1.69 (0.57 to 4.96)
Probiotics vs. Control
0.98 (0.16 to 6.15)
Synbiotics vs. Control
0.1 0.5 1.0 1.5 2.0 2.5 3.0 3.5 4.0 4.5 5.0 5.5 6.0 6.5
Risk ratio

eFigure 5. Comparison-adjusted funnel plots

Each point represents a study with different colors, indicating different treatment comparisons. The x-axis shows the comparison-adjusted effect size (ES_adj), and the y-axis indicates the standard error.

ICU intensive care unit; *Ctrl* control; *Pre* prebiotics; *Pro* probiotics; *Syn* synbiotics; *VAP* ventilator-associated pneumonia

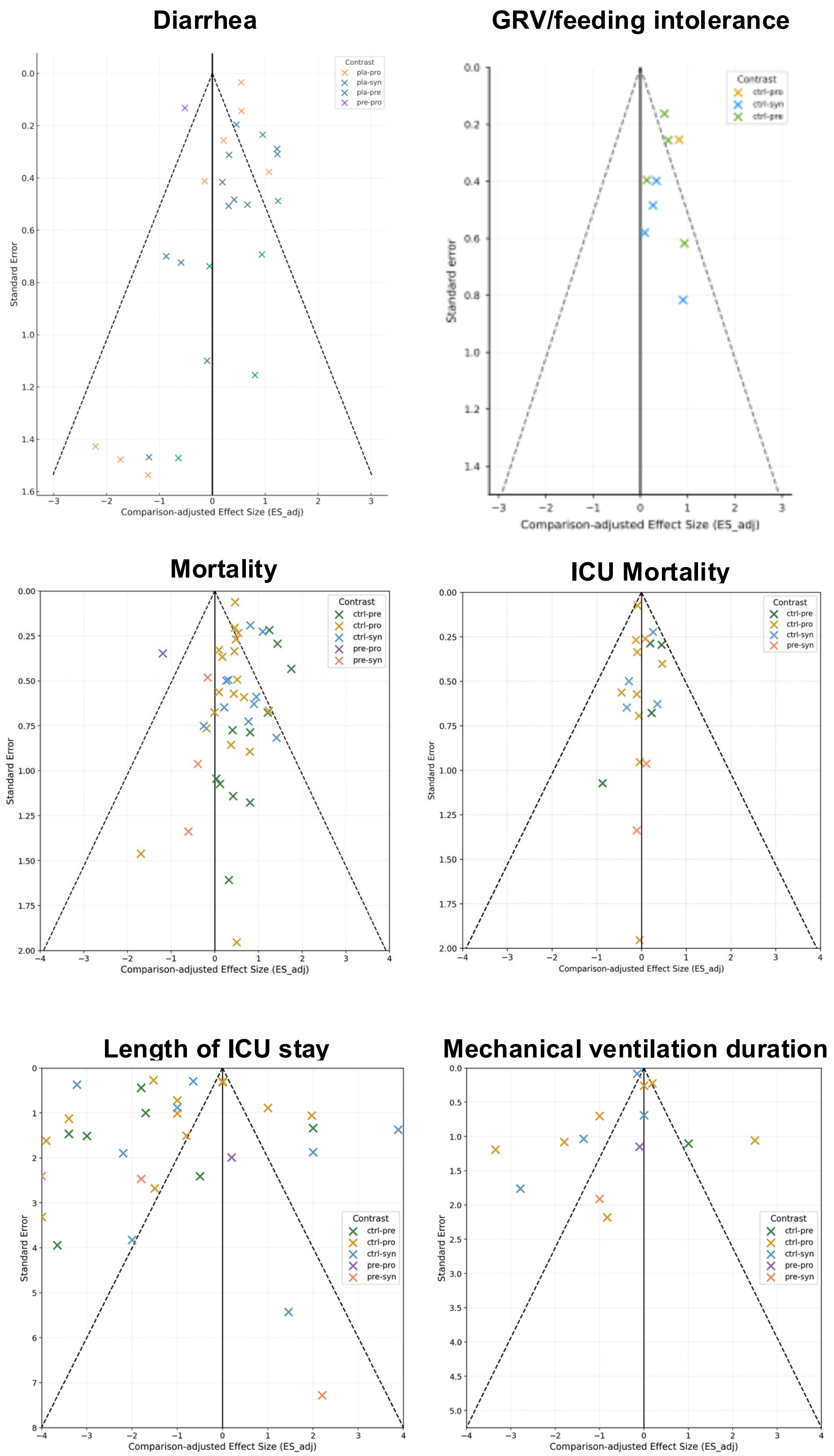

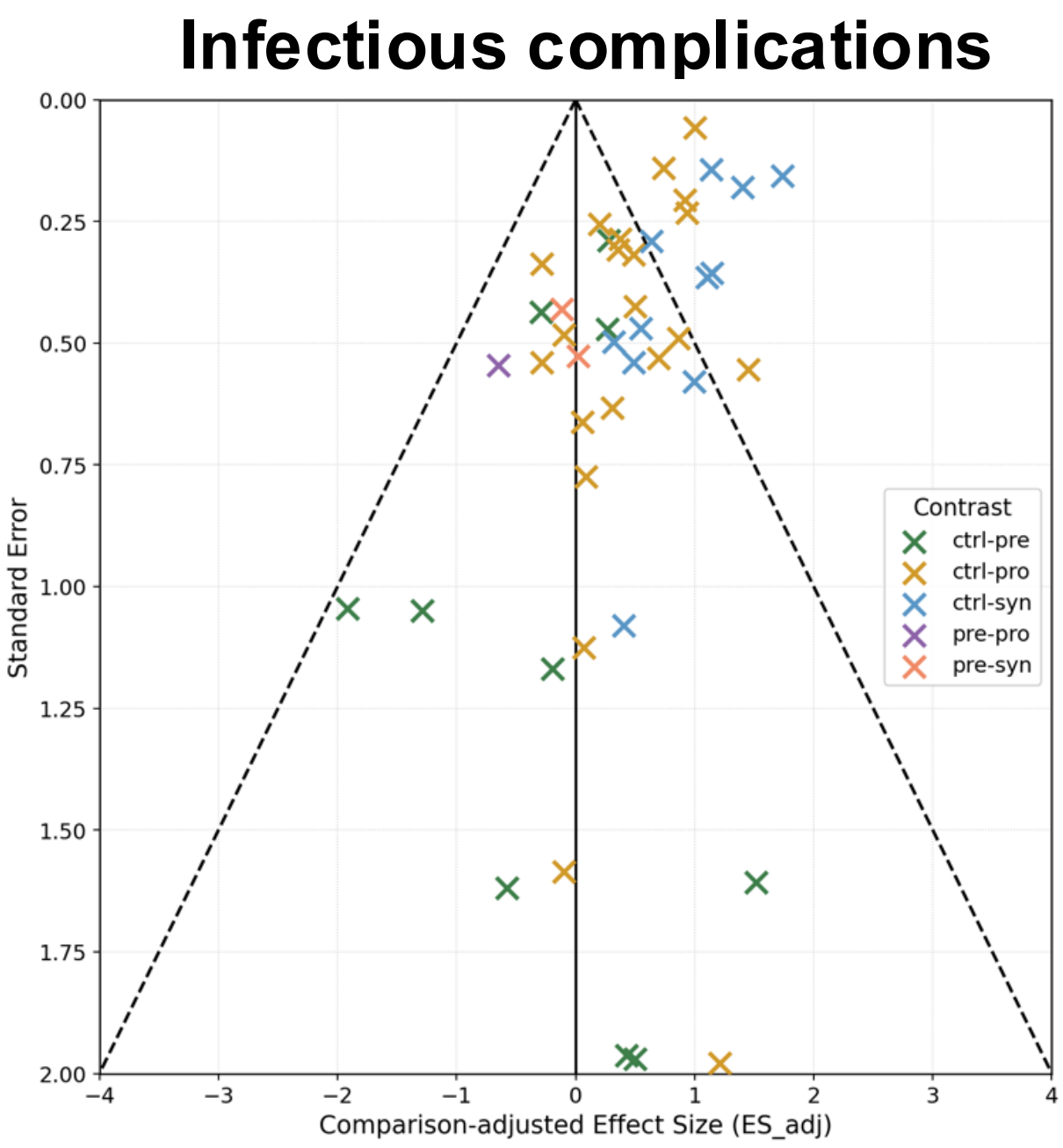

Infectious complications
Standard Error
Comparison-adjusted Effect Size (ES_adj)
Contrast
ctrl-pre
ctrl-pro
ctrl-syn
pre-pro
pre-syn

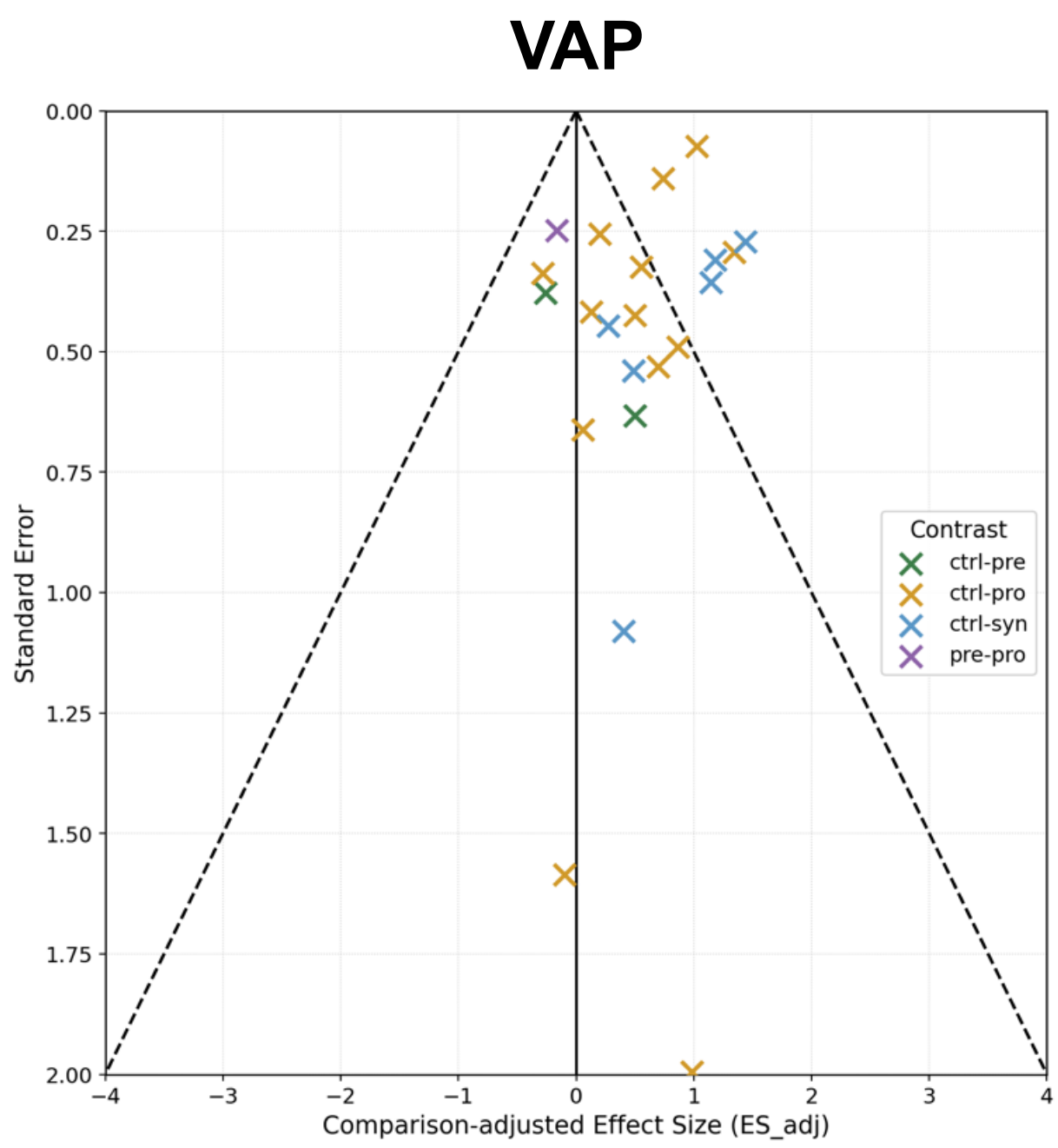

VAP
Standard Error
Comparison-adjusted Effect Size (ES_adj)
Contrast
ctrl-pre
ctrl-pro
ctrl-syn
pre-pro

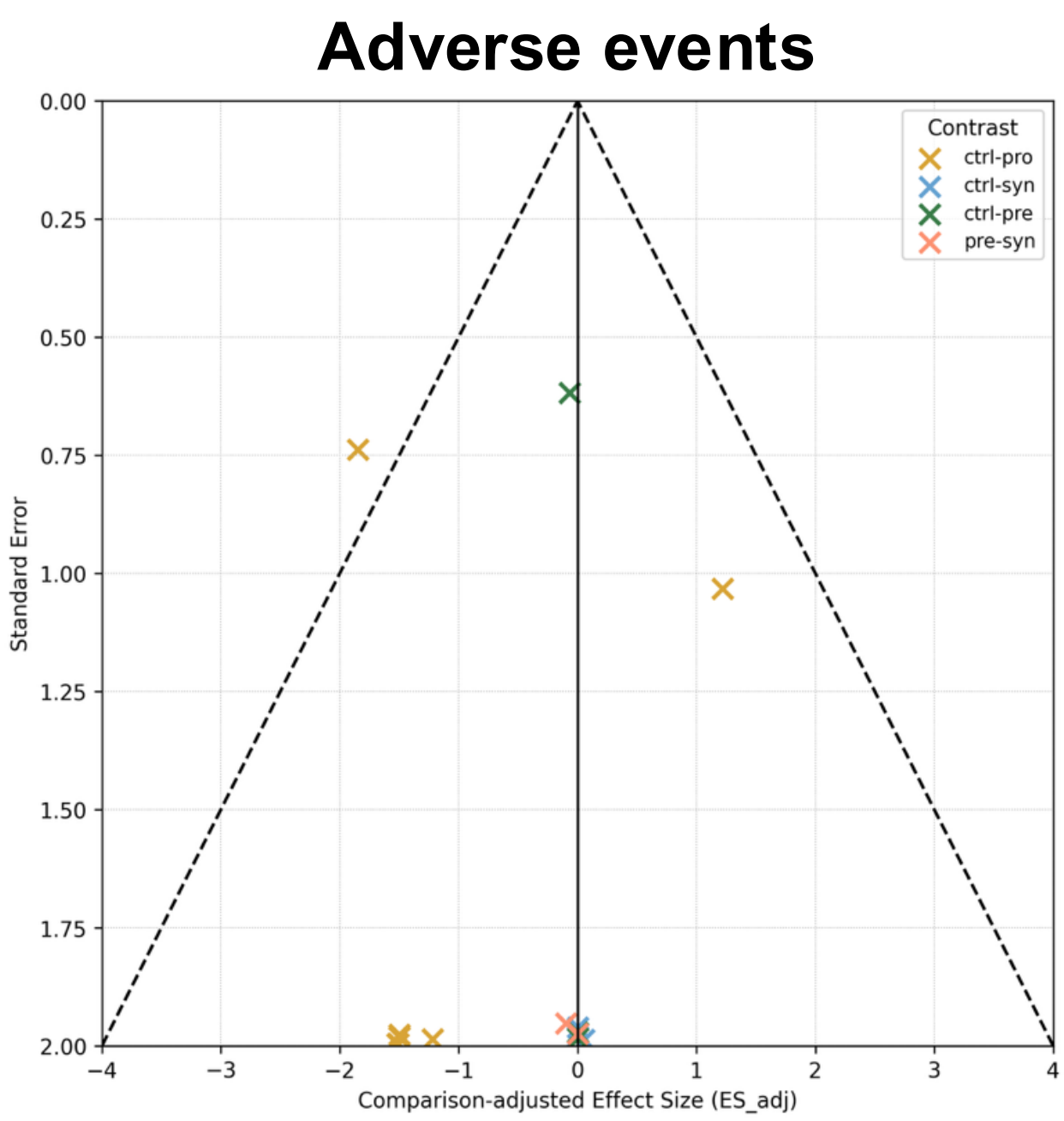

Adverse events
Standard Error
Comparison-adjusted Effect Size (ES_adj)
Contrast
ctrl-pro
ctrl-syn
ctrl-pre
pre-syn

eFigure 6. Network map for subgroups and sensitivity analyses of gastrointestinal symptoms

Network plots illustrating the map of treatment comparisons for subgroup analyses (surgical patients, internal medicine patients, intervention period of 7–13 days, and oligosaccharide addition) and sensitivity analyses (exclusion of studies at high risk of bias, exclusion of small studies with fewer than 50 participants, and restriction to a limited control group) for gastrointestinal symptoms. The limited control group comprised trials in which the comparator was placebo or usual care explicitly confirmed to be fiber-free. The node size is proportional to the number of patients randomized to each intervention, and the edge thickness is proportional to the number of studies for each comparison.

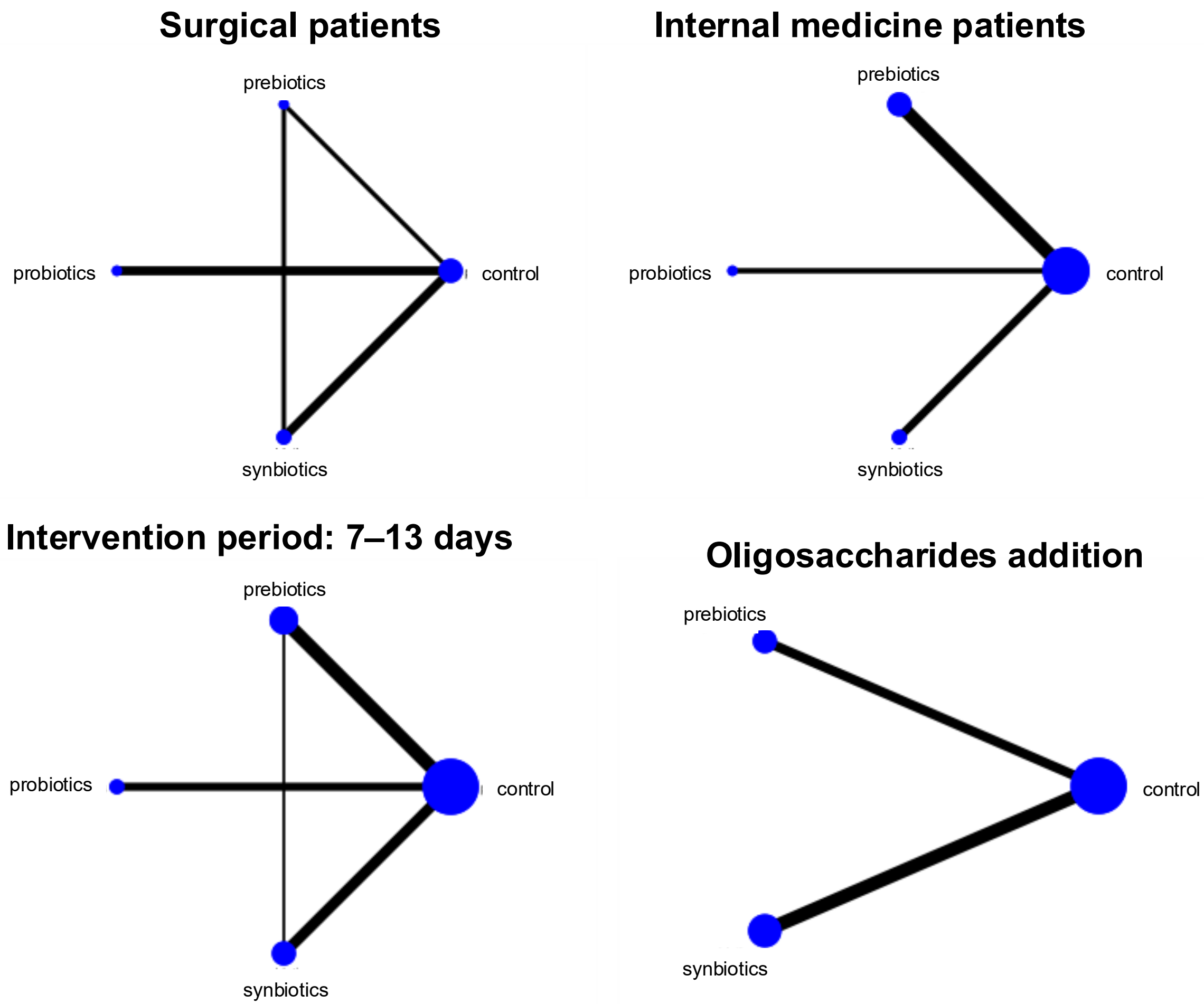

High-Risk Bias Study Exclusion

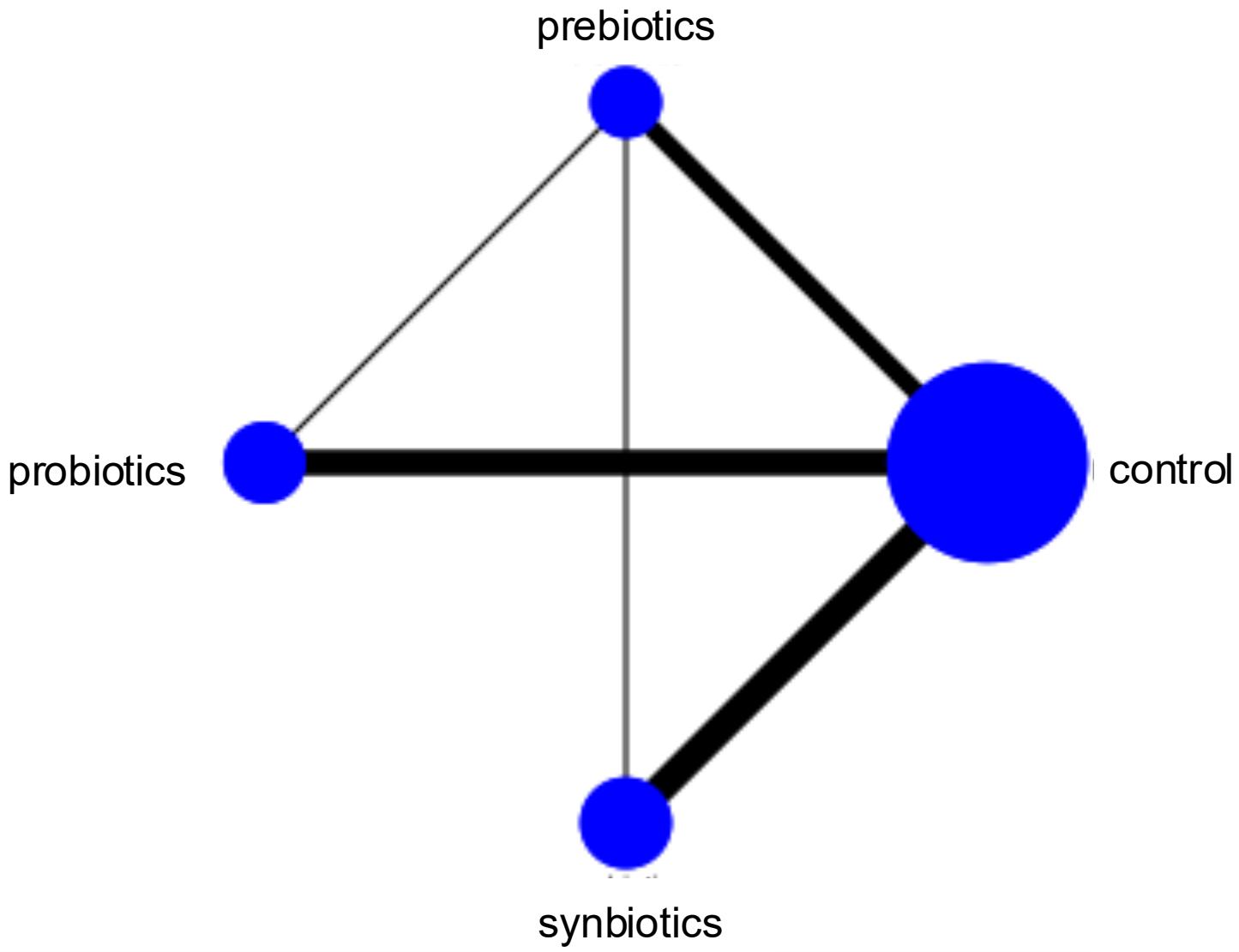

Excluding small studies (<50 participants)

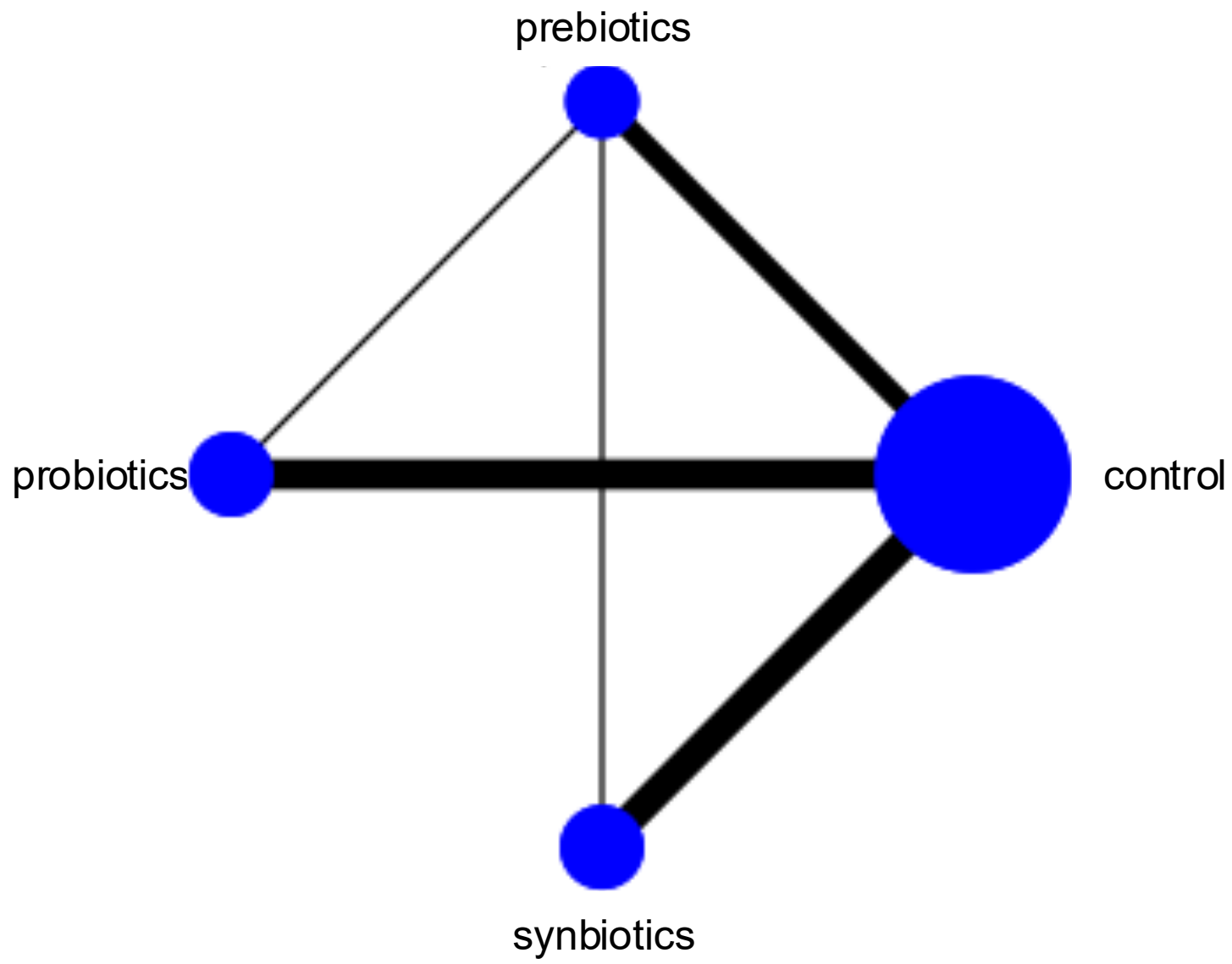

Limited control group

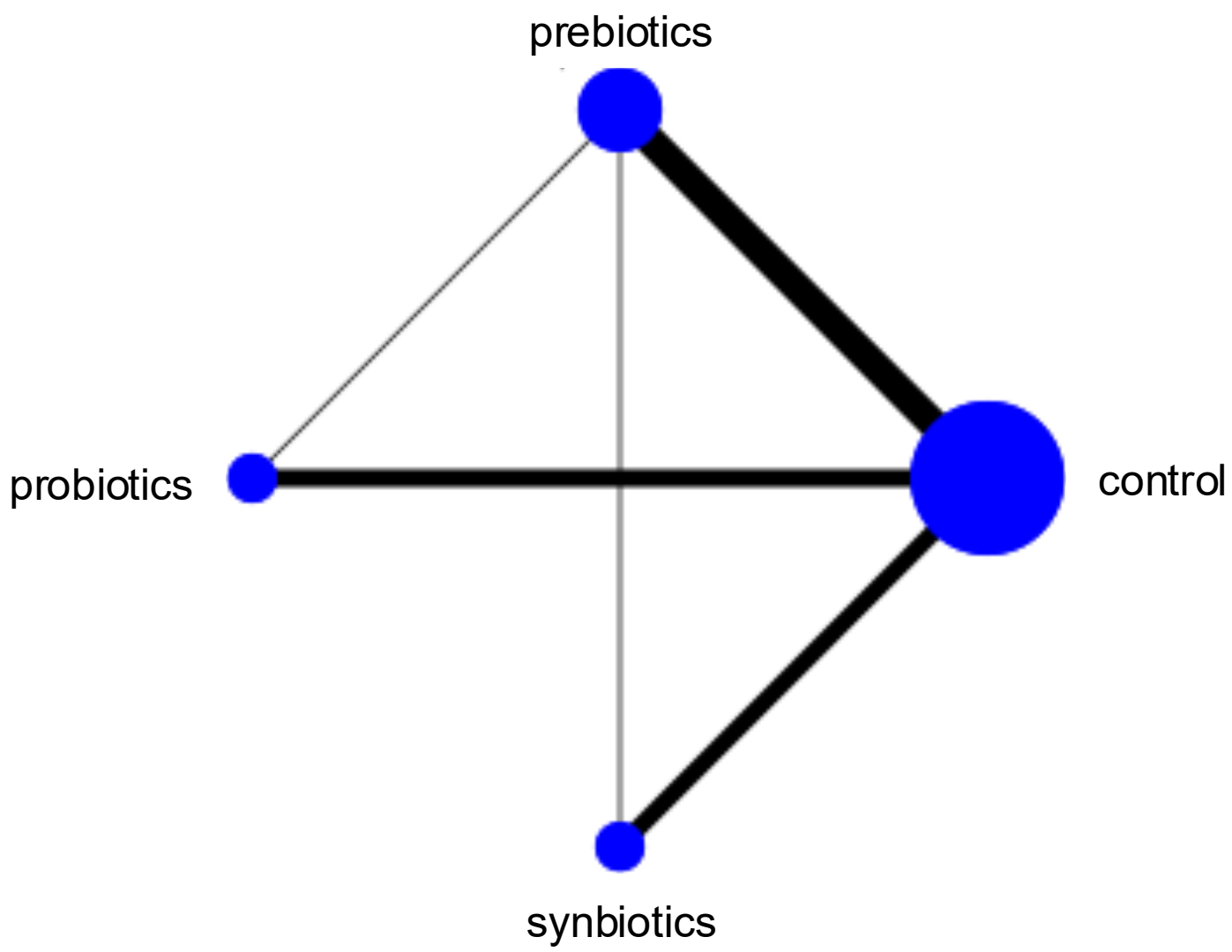

eFigure 7. Forest plot for subgroup analysis and sensitivity analysis of gastrointestinal symptoms

Forest plots illustrating the results of network meta-analysis for gastrointestinal symptoms in subgroup analyses (surgical patients, internal medicine patients, intervention period of 7–13 days, and oligosaccharide addition) and sensitivity analyses (exclusion of studies at high risk of bias, exclusion of small studies with fewer than 50 participants, and restriction to a limited control group). The limited control group comprised trials in which the comparator was placebo or usual care explicitly confirmed to be fiber-free. Results are expressed as risk ratios with 95% confidence intervals (CIs) compared to the control; values <1 indicate a reduction in gastrointestinal symptoms.

Ctrl control, *Pre* prebiotics, *Pro* probiotics, *Syn* synbiotics

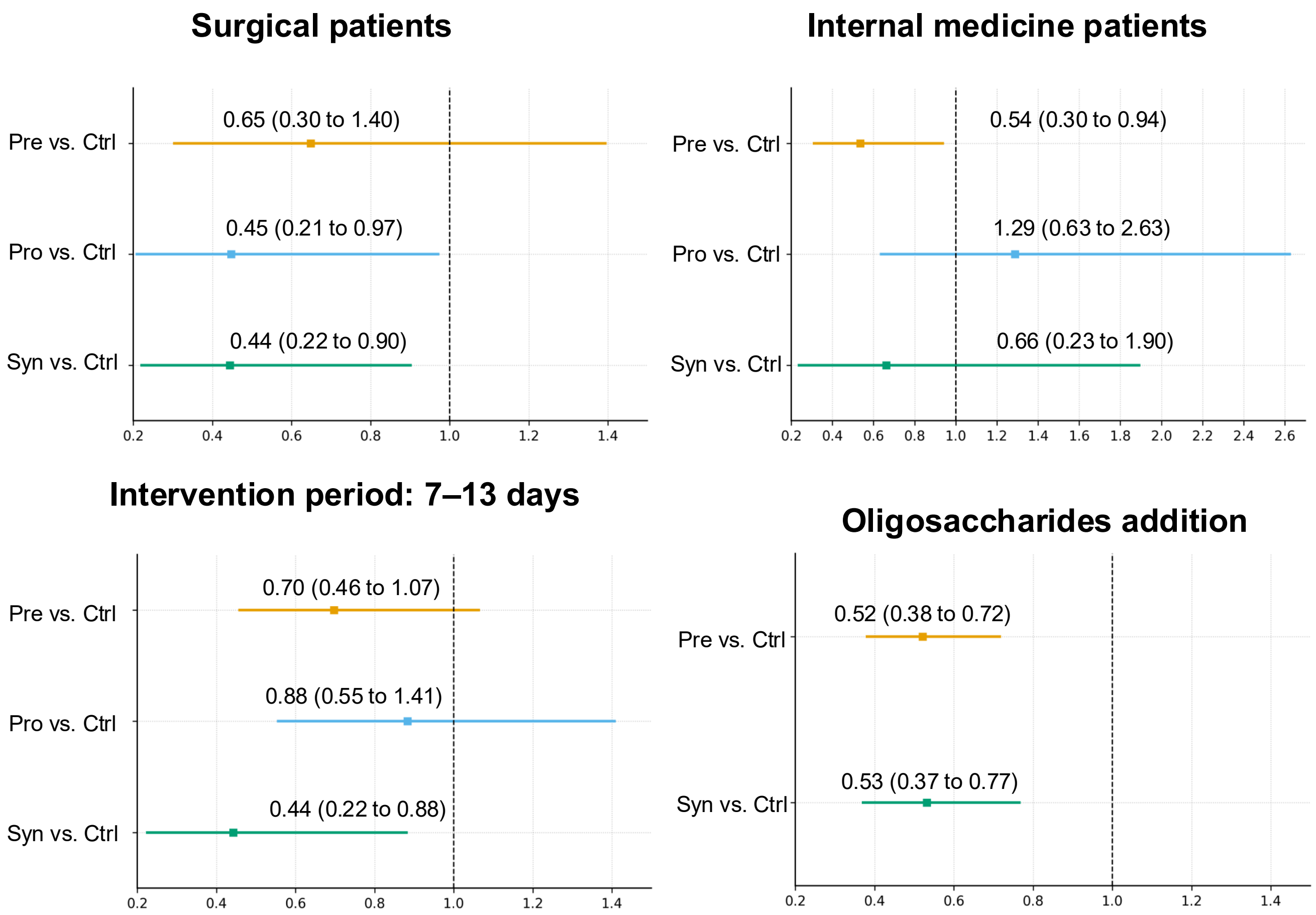

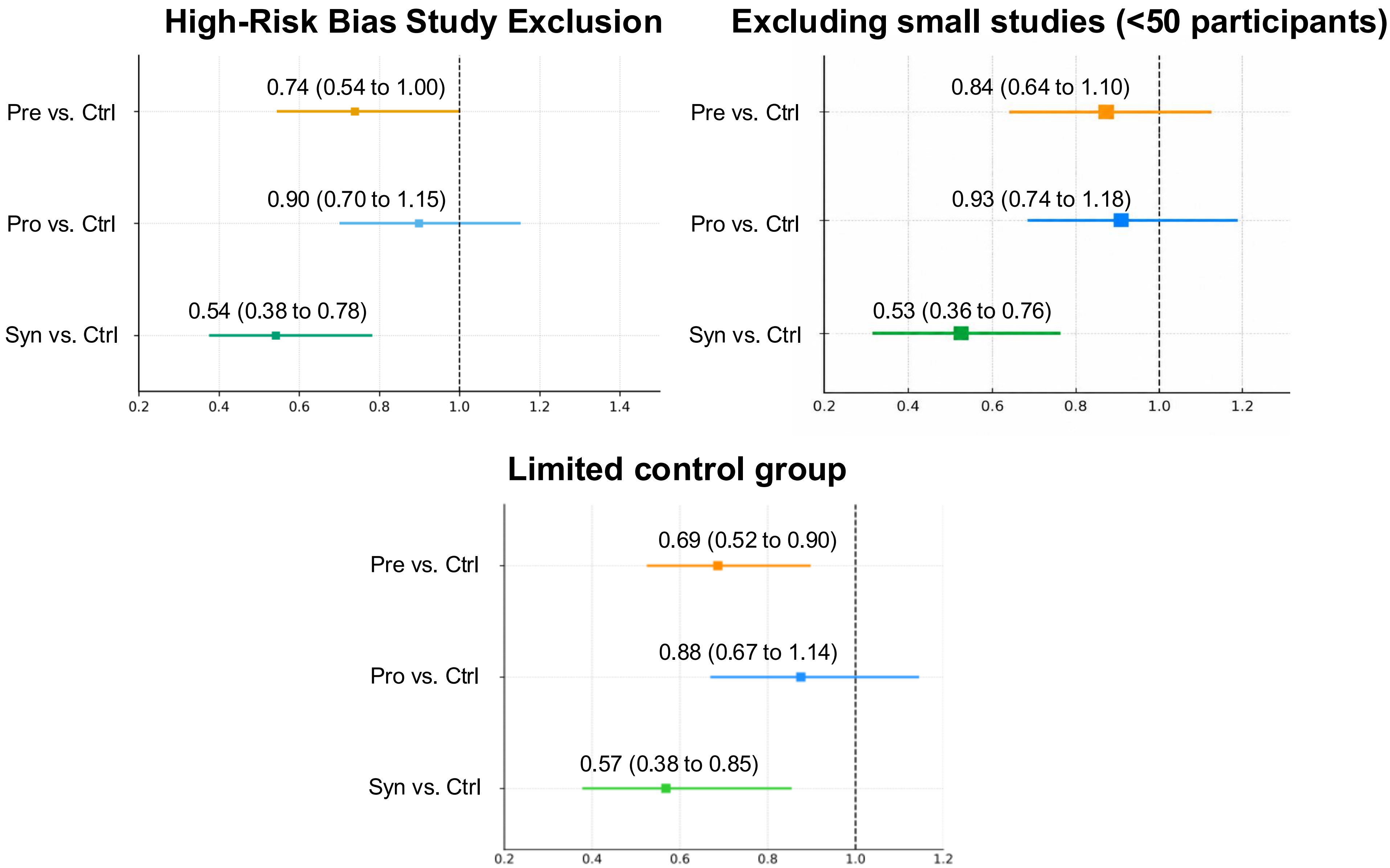
High-Risk Bias Study Exclusion
Pre vs. Ctrl
0.74 (0.54 to 1.00)
Pro vs. Ctrl
0.90 (0.70 to 1.15)
Syn vs. Ctrl
0.54 (0.38 to 0.78)
0.2
0.4
0.6
0.8
1.0
1.2
1.4
Excluding small studies (<50 participants)
Pre vs. Ctrl
0.84 (0.64 to 1.10)
Pro vs. Ctrl
0.93 (0.74 to 1.18)
Syn vs. Ctrl
0.53 (0.36 to 0.76)
0.2
0.4
0.6
0.8
1.0
1.2
Limited control group
Pre vs. Ctrl
0.69 (0.52 to 0.90)
Pro vs. Ctrl
0.88 (0.67 to 1.14)
Syn vs. Ctrl
0.57 (0.38 to 0.85)
0.2
0.4
0.6
0.8
1.0
1.2

eFigure 8. Comparison-adjusted funnel plots for subgroups and sensitivity analyses of gastrointestinal symptoms

Comparison-adjusted funnel plot assessing the small-study effects of gastrointestinal symptoms. Each point represents a study with different colors, indicating different treatment comparisons. The x-axis shows the comparison-adjusted effect size (ES_adj), and the y-axis indicates the standard error.

Ctrl control, *Pre* prebiotics, *Pro* probiotics, *Syn* synbiotics

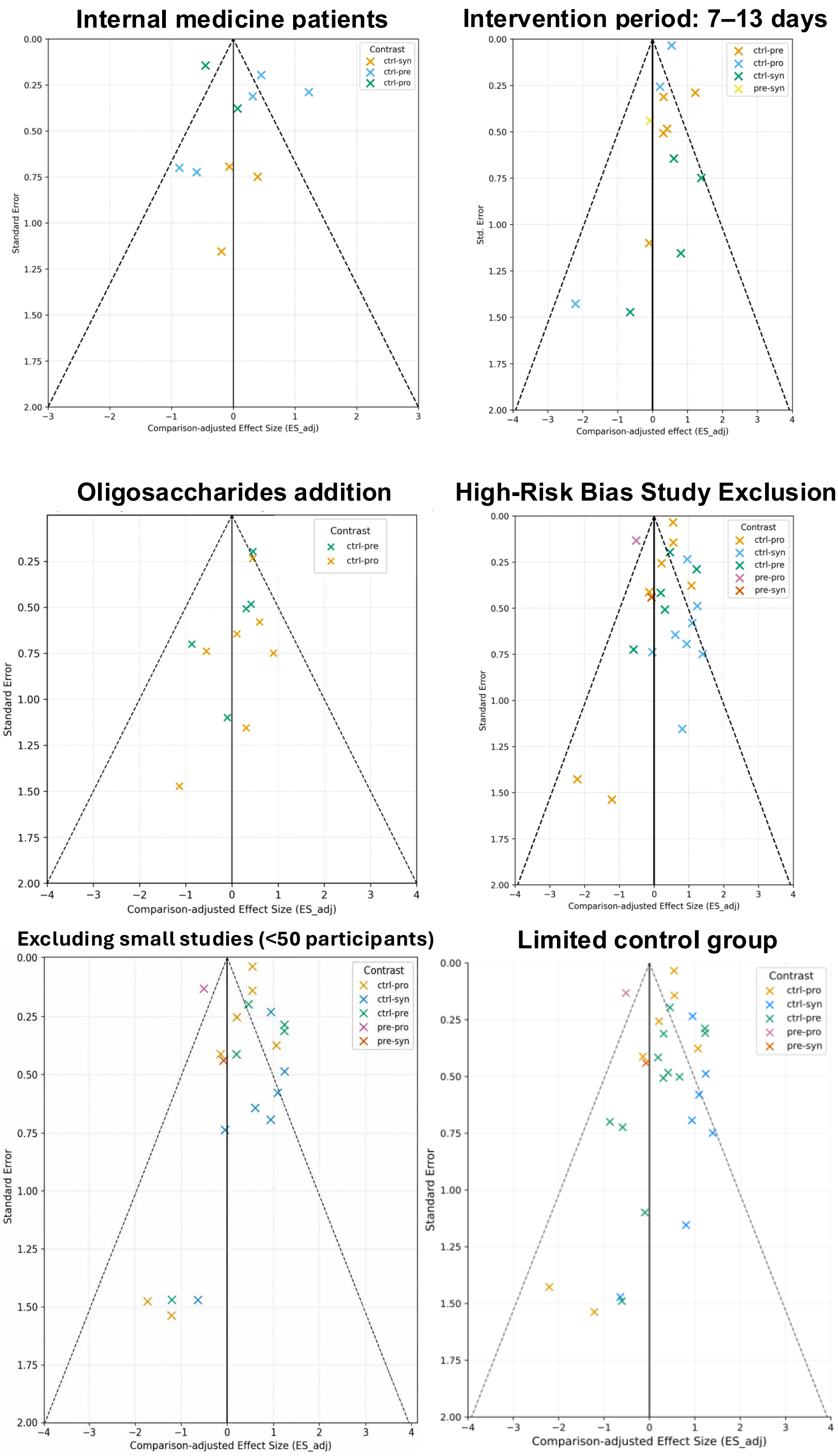

Supplement: Supplementary file 1 [file mmc1.pdf]
